# Bimolecular Excited-State Proton-Coupled Electron Transfer within Encounter Complexes

**DOI:** 10.1021/jacs.2c10165

**Published:** 2023-02-20

**Authors:** Kristina Martinez, Sydney M. Koehne, Kaitlyn Benson, Jared J. Paul, Russell H. Schmehl

**Affiliations:** † Department of Chemistry, Tulane University, New Orleans, Louisiana 70118, United States; ‡ Department of Chemistry, Villanova University, Philadelphia, Pennsylvania 19085, United States; § Department of Chemistry, Northwestern University, Evanston, Illinois 60208, United States; ∥ Division of Chemistry and Chemical Engineering, Caltech, Pasadena, California 91125, United States

## Abstract

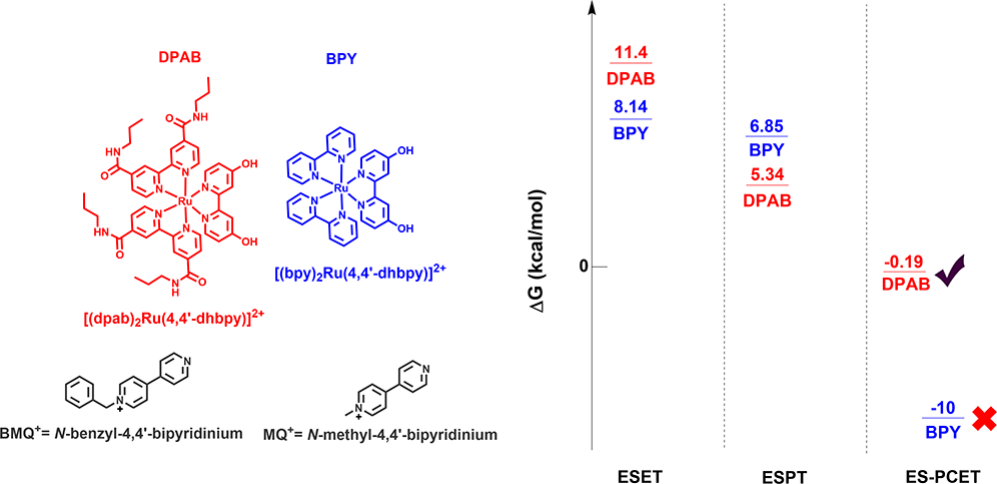

Bimolecular excited-state proton-coupled electron transfer (PCET*) was observed for reaction of the triplet MLCT state of [(dpab)_2_Ru(4,4′-dhbpy)]^2+^ (dpab = 4,4′-di(*n*-propyl)amido-2,2′-bipyridine, 4,4′-dhbpy = 4,4′-dihydroxy-2,2′-bipyridine) with *N*-methyl-4,4′-bipyridinium (MQ^+^) and *N*-benzyl-4,4′-bipyridinium (BMQ^+^) in dry acetonitrile solutions. The PCET* reaction products, the oxidized and deprotonated Ru complex, and the reduced protonated MQ^+^ can be distinguished from the excited state electron transfer (ET*) and the excited state proton transfer (PT*) products by the difference in the visible absorption spectrum of the species emerging from the encounter complex. The observed behavior differs from that of reaction of the MLCT state of [(bpy)_2_Ru(4,4′-dhbpy)]^2+^ (bpy = 2,2′-bipyridine) with MQ^+^, where initial ET* is followed by diffusion-limited proton transfer from the coordinated 4,4′-dhbpy to MQ^0^. The difference in observed behavior can be rationalized based on changes in the free energies of ET* and PT*. Substitution of bpy with dpab results in the ET* process becoming significantly more endergonic and the PT* reaction becoming somewhat less endergonic.

## Introduction

The utilization of solar energy to drive chemical reactions is of increasing value. Solar light driven transformations have the advantage of exploiting the generation of electronic excited states through light absorption to drive reactions that, in the ground state, are endergonic.^1^ Current work in the field of light-to-chemical conversion is in pursuit of generating viable, storable fuels from abundant substrates such as CO_2_ and H_2_O.^2^ Reactions such as the reduction of carbon dioxide require multiple electron *and* proton transfer events. These multielectron, multiproton reactions are facilitated by proton-coupled electron transfer (PCET) in natural systems such as water oxidation in photosynthesis.^3^ The advantage of PCET over sequential redox and acid–base (electron transfer-proton transfer (ET/PT) or proton transfer-electron transfer (PT/ET)), is that PCET avoids high activation barrier steps often associated with the sequential reaction pathway.^4^

Incorporating light absorption and photoexcited species into PCET reaction schemes creates a system poised to take light energy and transform it into new chemical bonds. The inclusion of light into these reaction schemes has been done in one of two ways, either through direct involvement of the excited state in the PCET reaction or the use of the light absorber to initiate electron transfer (ET) reactions through an oxidative or reductive quenching pathway. In a reaction where the excited state is involved directly in PCET, the light absorber can act as either a proton and electron donor or acceptor. A recent review of such reactions was compiled by Dempsey and co-workers.^5^

Focusing on reactions that involve light absorbers that act as both proton and electron donors, systems that have been investigated to date often involve the use of covalently linked chromophores and substrates, or the use of preformed hydrogen-bond bridged donor and acceptor complexes.^6−8^ One example of a such a system, published by Wenger and co-workers, involves a cyclometalated Ir complex incorporating a biimidazole ligand as a proton donor.^9^ In this work, the biimidazole ligand forms a salt bridge to the electron acceptor, dinitrobenzoate. The work uncovered that through tethering the donor and acceptor together via hydrogen bonds, the rate of ET was enhanced when compared to the *N*-methylated biimidazole complex. Additional examples of excited-state proton and electron donors can be seen in work done by Nocera and Wenger, reporting systems involving ruthenium diimine complexes and porphyrins as light absorbing molecules with the ability to partake in electron and proton transfer reactions. These systems provide elegant examples of excited state processes involving ET and PT, but lack definitive spectral evidence that distinguishes ET/PT from PCET.

**Figure 1:**
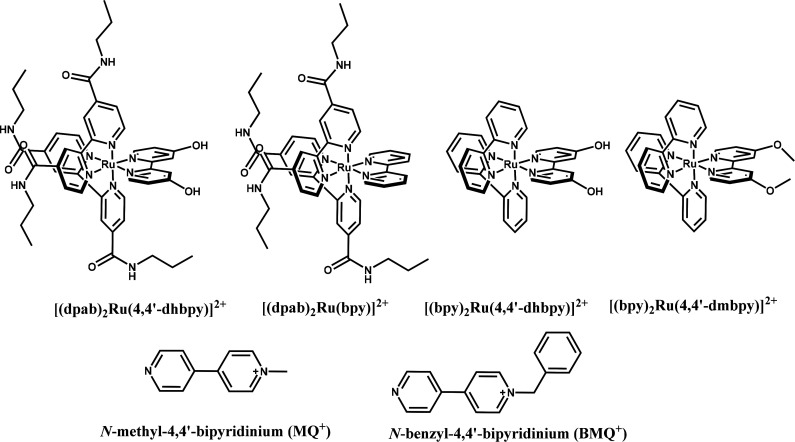
Structures of complexes and quenchers studied in this series.

To generate systems for which clear observation of concerted electron and proton transfer reaction from excited states is possible, we expanded on earlier work of the Meyer group that employed Ru^II^ hydroxyphenanthroline complex electron/proton donors and monomethylated 4,4′-bipyridinium electron/proton acceptors (MQ^+^, Figure 1). These systems make use of transient spectrophotometry to unambiguously distinguish excited state reactions that are PT, ET, and PCET. In work similar to the original Meyer paper, we reported a system that utilized a Ru^II^ diimine complex bearing a hydroxylated bipyridine ligand and MQ^+^. In this system, we explored the thermodynamic landscape of reactions.^10−12^ We discovered that despite the favorability of a concerted PCET reaction from the excited-state potential energy surface, outer sphere electron transfer was observed. We speculated that because of a combination of electrostatic repulsion from the same charge species and a small driving force for proton transfer, and therefore also weak hydrogen bonding, the otherwise favorable concerted excited-state PCET (PCET*) reaction was not observed.

For this work, we have synthesized a derivative of the aforementioned [(bpy)_2_Ru(4,4′-dhbpy)]^2+^ that contains electron withdrawing amido substituted ancillary ligands that should serve to enhance the excited-state acidity, while simultaneously making electron transfer from the excited state of this chromophore more endergonic: [(dpab)_2_Ru(4,4′-dhbpy)]^2+^ (dpab = 4,4′-dipropylamido-2,2′-bipyridine); Figure 1). We also report here on the use of *N*-benzyl-4,4′-bipyridinium (BMQ^+^) as a proton and electron acceptor for both reaction with [(dpab)_2_Ru(4,4′-dhbpy)]^2+^ and [(bpy)_2_Ru(4,4′-dhbpy)]^2+^. The systems were investigated spectroscopically using nanosecond transient absorption spectroscopy, and the thermodynamic parameters were determined by a combination of spectroscopic and electrochemical techniques. Herein we report on the mechanisms for excited state reduction and protonation of the two monoquats.

## Experimental Section

### Materials

The following reagents were purchased and used without further purification: 4,4′-bipyridine (Acros Organics), benzyl bromide (Alfa), methyl iodide (Sigma-Aldrich), 4-bromobenzenediazonium tetrafluoroborate (Alfa), Di-μ-chlorobis[(*p*-cymene)chlororuthenium(II)] (Strem), 2,2′-bipyridine (Sigma-Aldrich), ammonium hexafluorophosphate (Oakwood). Acetonitrile was distilled from CaH_2_ prior to use. Tetrabutylammonium hexafluorophosphate (TBAPF_6_) (TCI America) was recrystallized from hot ethanol, filtered, and dried in vacuo prior to use. 4,4′-Bis(dipropylamido)-2,2′-bipyridine (dpab), 4,4′-dihydroxy-2,2′-bipyridine (4,4′-dhbpy), *N*-methyl-4,4′-bipyridinium hexafluorophosphate (MQ^+^), and [(bpy)_2_Ru(4,4′-dhbpy)](PF_6_)_2_ were synthesized following previously reported literature procedures.^10,13,14^ Deuterated solvents (methanol-*d*_4_ and acetonitrile-*d*_3_) were purchased from Cambridge Isotope Laboratories (CIL). CD_3_CN was dried over 3 Å molecular sieves prior to use in kinetic isotope studies.

### Synthesis

#### [(*p*-Cymene)Ru(4,4′-dhbpy)Cl]Cl

[(*p*-Cymene)Ru(4,4′-dhbpy)Cl]Cl was prepared via modification of a previously reported synthesis for an analogous compound.^15^ [(*p*-Cymene)RuCl_2_]_2_ (0.327 g, 0.53 mmol) and 4,4′dihydroxy-2,2′-bipyridine (0.201 g, 1.06 mmol) were added to 20 mL acetonitrile and degassed for 20 min prior to refluxing for 4 h under nitrogen atmosphere. During reflux a yellow precipitate formed. Upon cooling, the solution was filtered, and the product was rinsed several times with acetonitrile. The crude product was then dissolved in methanol and the solution was filtered to remove any undissolved material. The product was reprecipitated using diethyl ether. The yield was 0.479 g (90.7%).

#### [(*p*-Cymene)Ru(bpy)Cl]Cl

[(*p*-Cymene)Ru(bpy)Cl]Cl was prepared in the same manner as above using 2,2′-bipyridine (0.259 g, 1.66 mmol) in place of 4,4′-dihydroxybipyridine and [(*p*-cymene)RuCl_2_]_2_ (0.507 g, 0.827 mmol). The product yield was 0.50 g (65%).

#### [(dpab)_2_Ru(4,4′-dhbpy)](PF_6_)_2_

[(*p*-Cymene)Ru(4,4′-dhbpy)Cl]Cl (0.100 g, 0.201 mmol) and 4,4′-bis(dipropylamido)-2,2′-bipyridine (0.132 g, 0.404 mmol) were dissolved in 5 mL amine-free *N*,*N*-dimethylformamide (dried over 3 Å molecular sieves). The mixture was degassed for 20 min prior to refluxing for 4 h under nitrogen atmosphere. Once cooled, excess acetone was added, and the mixture was cooled in the freezer overnight. The precipitated product, [(dpab)_2_Ru(4,4′-dhbpy)]Cl_2_, was collected on a fine fritted filter. The complex was precipitated as the hexafluorophosphate salt by addition of a molar excess of aqueous ammonium hexafluorophosphate to an aqueous solution of the product. [(dpab)_2_Ru(4,4′-dhbpy)](PF_6_)_2_ was purified to remove the predominant impurity [Ru(dpab)_3_](PF_6_)_2_ using column chromatography. The crude product was added to an alumina column with an eluent of 5% methanol in dichloromethane. The desired product remained unmovable with this eluent mixture, but the [Ru(dpab)_3_](PF_6_)_2_ was quickly removed from the column. A secondary eluent mixture was added, 1:1 H_2_O and acetonitrile. The desired product was successfully removed from the column yielding 0.070 g (28%). The product was characterized by ^1^H NMR, ^13^CNMR, COSY, and ESI-MS (Figure.S1). ^1^H NMR (300 MHz, CD_3_CN, residual internal (CD_2_H)CN δ 1.94 ppm) δ 8.91 (s, 4H), 7.98 (d, *J* = 5.9 Hz, 2H), 7.82 (d, *J* = 6.0 Hz, 2H), 7.74 (d, *J* = 5.8 Hz, 2H), 7.70–7.59 (m, 6H), 7.52 (s, 2H), 7.17 (d, *J* = 6.4 Hz, 2H), 6.72 (d, *J* = 6.5 Hz, 2H), 3.37 (m, *J* = 14.4, 6.8 Hz, 9H), 1.62 (m, *J* = 7.5 Hz, 8H), 0.95 (q, *J* = 7.1 Hz, 12H).

#### [(dpab)_2_Ru(bpy)](PF_6_)_2_

The above synthesis was repeated using the aforementioned procedure for [(dpab)_2_Ru(4,4′-dhbpy)](PF_6_)_2_ using [(*p*-cymene)Ru(bpy)Cl]Cl (0.152 g, 0.329 mmol) and 4,4′-bis(dipropylamido)-2,2′-bipyridine (0.214 g, 0.656 mmol). For this complex, no column was used to treat the product, because the presence of [Ru(dpab)_3_](PF_6_)_2_ in trace amounts had no effect on subsequent experiments. The yield was 0.091 g (23%). The product was characterized by ^1^H NMR (Figure S1), ^13^C NMR, COSY, and ESI-MS (Figure S2). ^1^H NMR (300 MHz, CD_3_CN, residual internal (CD_2_H)CN δ 1.94 ppm) δ 8.95 (t, *J* = 1.9 Hz, 4H), 8.54 (dd, *J* = 8.1, 1.2 Hz, 2H), 8.12 (td, *J* = 7.9, 1.5 Hz, 2H), 7.88 (t, *J* = 5.5 Hz, 4H), 7.72 (ddt, *J* = 7.9, 4.1, 1.9 Hz, 6H), 7.53 (d, *J* = 5.1 Hz, 4H), 7.44 (ddd, *J* = 7.2, 5.6, 1.3 Hz, 2H), 3.40 (dtd, *J* = 7.7, 6.1, 2.1 Hz, 8H), 1.66 (hd, *J* = 7.3, 2.2 Hz, 8H), 0.98 (td, *J* = 7.4, 2.1 Hz, 12H).

#### *N*-Benzyl-4,4′-bipyridinium

4,4′-Bipyridine (5.04 g, 0.0323 mol) and benzyl bromide (3.8 mL, 0.032 mol) were dissolved in toluene and refluxed for 12 h under argon atmosphere. A yellow precipitate was isolated by filtration and washed with diethyl ether. The crude product, *N*-benzyl-4,4′-bipyridinium bromide (BMQ^+^), was dissolved in water and precipitated out as a hexafluorophosphate salt by the addition of a molar excess of aqueous ammonium hexafluorophosphate. BMQ^+^ was purified by recrystallization from hot ethanol. The product was collected and dried in vacuo to yield 5.397 g (51.5%). The compound was characterized by ^1^H NMR and ^13^CNMR. ^1^H NMR (300 MHz, CD_3_CN, residual internal (CD_2_H)CN δ 1.94 ppm) δ 8.84 (dp, *J* = 7.2, 2.7, 2.2 Hz, 4H), 8.35–8.25 (m, 2H), 7.83–7.74 (m, 2H), 7.49 (s, 5H), 5.76 (s, 2H). The p*K*_a_ of HBMQ^2+^ was measured by titration monitoring the equilibrium by ^1^H NMR.

### Absorption and Emission Spectroscopy

UV–vis absorption spectra were collected on either an HP 8452 Diode Array spectrophotometer, or an Ocean Optics HR2000+ES. For UV–visible absorption spectra, a 1 cm path length cell, l, was used. Emission spectra were collected using a PTI Quantamaster spectrophotometer equipped with a red sensitive Hammammatsu R928 PMT detector or using an Ocean Optics HR2000+ES CCD detector. Unless otherwise stated, absorption and emission spectra were collected in N_2_ or Ar degassed acetonitrile solution.

### Determination of p*K*_a_ Values

The p*K*_a_ values for ground-state complexes were determined by a photometric titration in acetonitrile solution. This technique employed the use of a base in acetonitrile for which the p*K*_a_ of the conjugate acid is known. The spectral differences between the protonated and deprotonated complex, at a wavelength where neither the reference base nor its conjugate acid absorb, allow for the determination of the equilibrium constant for the proton exchange in acetonitrile. The molar extinction coefficients were used in calculating the concentration of the protonated and deprotonated ruthenium complex. These were obtained from UV–vis absorbance spectra of solutions with a known concentration of ruthenium.

### Electrochemistry and Spectroelectrochemistry

Electrochemical and spectroelectrochemical measurements were carried out using a CH Instruments 630E Electrochemical Analyzer/Workstation. All measurements were done in acetonitrile dried over CaH_2_ and distilled before use. Unless otherwise stated, cyclic voltammetric measurements were done using a glassy carbon working electrode, a platinum wire counter electrode, and an Ag wire pseudoreference electrode, and ferrocene as an internal standard. Spectroelectrochemical measurements were made using an Ocean Optics HR2000 spectrophotometer along with a Pine Research Instruments platinum honeycomb working electrode, a Pt wire counter electrode, and a Ag/AgCl reference electrode.

### General Nanosecond Transient Absorption (TA) Procedures

Nanosecond transient absorption measurements were done on an Applied Photophysics LKS 60 Laser Flash Photolysis system with laser excitation from a Quantel Brilliant B Q-switched laser with second and third harmonic attachments and an OPO (OPOTEK) for visible light generation, and data recorded using an Agilent Infinium digitizer. Laser excitation of the sample was typically supplied at 450 nm, with a power output of 12 mJ/pulse. Spectra were typically corrected for emission by adding the absolute value of an emission decay signal (calculated as a transient absorbance signal) to the observed transient absorption signal at the same wavelength. Unfortunately, this resulted often in an overcompensation that led to observation of a net absorption signal, especially at wavelengths where the intensity of the amplified white light source of the TA was weak (>650 nm). Observed maxima in the red varied depending on the degree of overcompensation. To maintain constant ionic strength in quenching experiments, tetrabutylammonium hexafluorophosphate was added to all samples studied by transient absorption.

### Preparation of Samples for KIE Studies

Samples were prepared using previously published techniques.^11^ For preparation of the deuterated complexes, the anion was exchanged for [BArF_24_] (using sodiumtetrakis[3,5-bis(trifluoromethyl)phenyl]borate).^21^ The complex was then dissolved in methanol-*d*_4_ to exchange the protons for deuterium. The methanol-*d*_4_ was removed, and the complex was taken and stored in the glovebox until use. All samples were prepared in the glovebox under dry, inert conditions until measurements were made.

## Results and Discussion

### Thermochemistry of [(LL)_2_Ru(4,4′-dhbpy)]^2+^ Complexes and Monoquaternarized 4,4′-bipyridine Derivatives

In an earlier publication we reported the excited-state reaction between [(bpy)_2_Ru(4,4′-dhbpy)]^2+^ with MQ^+^.^11^ The thermochemical analysis revealed that both ET* and PT* were endergonic. Transient absorption studies indicated that the reaction proceeded through a multistep process in which ET* products could be identified as the initial cage escape products through comparison of the kinetics at wavelengths where the reduced, protonated quencher, HMQ^+^, absorbs strongly. Investigation of the effect of the concentration of [(bpy)_2_Ru(4,4′-dhbpy)]^2+^ on protonation of the ET* product, MQ^0^, indicated that the [(bpy)_2_Ru(4,4′-dhbpy)]^2+^ in solution (*not* the Ru^III^ complex) was responsible for the protonation of MQ^0^. This result prompted us to explore how changing the excited-state redox potential, such that electron transfer would be less likely to occur as the predominant reaction within the encounter complex, would influence the reaction mechanism and drive it closer to concerted proton and electron transfer within the cage.

Characterization of new Ru^II^ diimine chromophores that may serve as electron and proton donors requires determination of thermodynamic information for ground and excited state acidity in CH_3_CN as well as redox potentials for the protonated and deprotonated complex in the ground and excited state. Some of the data can be difficult to obtain experimentally, such as the p*K*_a_ of the Ru^III^ hydroxydiimine complex. However, reasonable estimates can be obtained from a variety of thermodynamic cycles. The results presented below describe experiments to obtain the required thermodynamic data for [(dpab)_2_Ru(4,4′-dhbpy)]^2+^ and the benzyl-4,4′-bipyridinium ion (BMQ^+^) in CH_3_CN.

#### Thermodynamic Parameters for [(dpab)_2_Ru(4,4′-dhbpy)](PF_6_)_2_

Details on the characterization of [(dpab)_2_Ru(4,4′-dhbpy)](PF_6_)_2_, including the absorption spectra, p*K*_a_ assessment, and cyclic voltammetry are presented in the Supporting Information. Because of the electron-withdrawing nature of the amide substituents on the ancillary ligands, it can be anticipated that the acidity of the ground and excited states will be increased relative to [(bpy)_2_Ru(4,4′-dhbpy)]^2+^ and that the Ru^III/II^ reduction will occur at a more positive potential, thereby making the complex more difficult to oxidize than the 2,2′-bipyridine complex.

By using cyclic voltammetry, the redox properties of [(dpab)_2_Ru(4,4′-dhbpy)](PF_6_)_2_ in acetonitrile solution were measured. The Ru^III/II^ potential was measured as 1.02 V vs Fc^+/0^; this is approximately 150 mV more positive than the previously studied [(bpy)_2_Ru(4,4′-dhbpy)](PF_6_)_2_, which occurs at 0.85 V vs Fc^+/0^ (ferrocinium/ferrocene internal reference).^10^ Reductive voltammetry of the complex reveals a complex series of reductions. Although the first reduction cathodic peak appears to be overlapping a second reduction wave, the potential can still be estimated as approximately −1.34 V vs Fc^+/0^ (Figure S3)

Measurement of the one-electron reduction potential of the *deprotonated* Ru^III^ complex is impeded by irreversibility in the cyclic voltammogram (see Figure S4). Despite this, the anodic peak potential associated with the Ru^III/II^ redox couple can be used as an estimate of the reduction potential giving a 410 mV difference in comparison to the fully protonated complex. The reductive voltammogram in the presence of one equivalent of base shows three cathodic peaks that are all irreversible.

**Figure 2:**
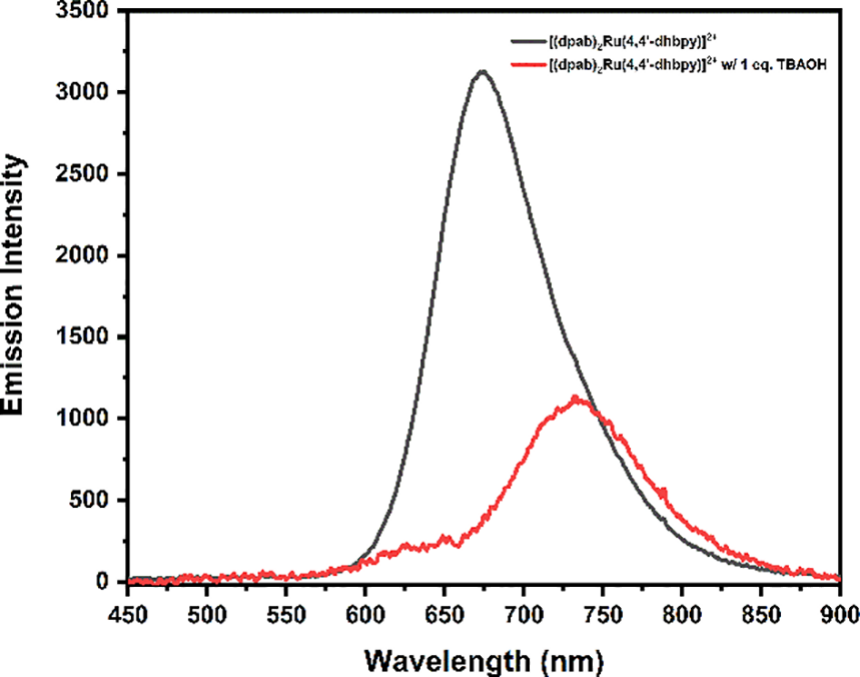
Emission spectrum of [(dpab)_2_Ru(4,4′dhbpy)]^2+^ and the monodeprotonated complex at −41 °C in acetonitrile.

The luminescence behavior of [(dpab)_2_Ru(4,4′-dhbpy)]^2+^ and the singly deprotonated complex in CH_3_CN at a temperature near the freezing temperature of CH_3_CN is shown in Figure 2. Both forms of the complex have emission maxima that are lower in energy than the bpy complex. Due to a combination of both a lower excited-state energy and more positive *E*^0^ Ru^III/II^ potential, [(dpab)_2_Ru(4,4′-dhbpy)](PF_6_)_2_ has an overall lower excited-state reducing potential than [(bpy)_2_Ru(4,4′-dhbpy)](PF_6_)_2_.^11^

Despite having less energy available for photoinduced electron transfer, the chromophore is a stronger photoacid. This change in acidity is due to the enhanced excited-state polarization toward the spectator ligands, as mentioned above. The more electropositive ruthenium center increases the sigma donation of electron density from the 4,4′-dhbpy ligand, thereby lowering the p*K*_a_ relative to the analogous bis-bipyridine complex.

The ground-state p*K*_a_ was measured by photometric titration with 2-aminobenzimidazole (p*K*_a_ (CH_3_CN) = 16.08).^16^ Using the calculated equilibrium constant for the proton exchange reaction in acetonitrile, the p*K*_a_ of [(dpab)_2_Ru(4,4′-dhbpy)]^2+^ was determined relative to 2-aminobenzimidazole to be 15.7.^11^ Since the monodeprotonated complex is nonemissive at room temperature and a polar aprotic solvent was used for these studies, a Förster thermodynamic cycle was used to calculate the excited-state p*K*_a_ (p*K*_a_*).^17^ The monodeprotonated complex was generated using a stoichiometric concentration of strong base (tetrabutylammonium hydroxide) in CH_3_CN. From here, the samples were cooled to just above the freezing point of acetonitrile. The p*K*_a_*, calculated using eq 1, is 12.4. This p*K*_a_ is 3 orders of magnitude more acidic than the ground-state, in line with reports on excited-state acidity for similar transition metal complexes.^1^ In eq 1, the *E*_0–0_ values needed for both the protonated and deprotonated complex were determined from the emission maximum taken from emission spectra collected in CH_3_CN at slightly above the freezing point of acetonitrile. This was done because at room temperature, the deprotonated complex does not have an observable emissive excited state. In order to generate the deprotonated complex, a stoichiometric amount of tetrabutyl ammonium hydroxide was used. It should be noted that the estimated value of *E*_0–0_ is almost certainly lower than the true *E*_0–0_, since the actual 0–0 transition of the emission spectrum will be obtained from the highest energy vibronic mode making up the emission spectrum. However, the spectra lacked a vibronic structure, preventing a thorough Franck–Condon analysis to obtain *E*_0–0_, so we opted for using the emission maxima. Since it is the difference in energy that matters (eq 1), we assumed the maxima would provide the most accurate measure of the difference.
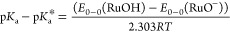
1The collected spectral, electrochemical, and titrimetric data for [(dpab)_2_Ru(4,4′-dhbpy)]^2+^ allowed determination of parameters for excited-state acidity and excited-state redox potential used to determine the free energy of excited-state electron transfer, proton transfer, and proton-coupled electron transfer in reaction with MQ^+^ and BMQ^+^.

The values for the excited-state redox potential (Ru^III/II*^) were calculated from the excited state energy and ground state Ru^III/II^ potential as with [(bpy)_2_Ru(4,4′-dhbpy)]^+^.^11^ The values are summarized in Table 1, along with other relevant photophysical properties of the complex.

**Table 1:** Selected Redox and Photophysical Data for [(dpab)_2_Ru(4,4′-dhbpy)]^2+^ and the Monodeprotonated Complex

	[(dpab)_2_Ru(4,4′-dhbpy)]^2+^	[(dpab)_2_Ru(4-O-4′-(OH)-bpy)]^+^	[(bpy)_2_Ru(4,4′-dhbpy)]^2+^^11^
*E*^0^(III/II), V vs Fc^+/0^	1.02	0.61	0.85
*E*^0^(III/II*), V vs Fc^+/0^	–0.82	–1.08	–1.05
*E*_em_, eV	1.87	1.69	1.93
λ_max,em_ (nm), –41 °C	665	734	642
τ_0_ (ns), 25 °C	615	<5	640
p*K*_a_ ± 0.02	17.5	–	17.7
p*K*_a_* ± 0.02	14.4	–	15.2

Compared to [(bpy)_2_Ru(4,4′-dhbpy)]^2+^, the dpab complex has a more positive oxidation potential and red-shifted emission, which couple together to generate a species more impervious to excited-state oxidation. However, because the excited-state is an order of magnitude more acidic than the photoexcited bpy complex, the possibility exists that excited-state proton transfer, or perhaps proton-coupled electron transfer, may occur.

#### Thermodynamic Parameters for BMQ^+^

The second quencher used in this study is a variation of the monomethylated 4,4′-bipyridinium quencher, *N*-benzyl-4,4′-bipyridinim (BMQ^+^). BMQ^+^ has two reversible one-electron reductions, the first at −1.20 V vs Fc^+/0^. Upon protonation with a sufficiently strong acid (0.2 M triflic acid), the reduction shifts to a more positive potential of −0.69 V vs Fc^+/0^. Both reduction of BMQ^+^ and HBMQ^+^ are more positive compared to MQ^+^ (−1.28 V vs Fc^+/0^) and HMQ^+^ (−0.75 V vs Fc^+/0^).

The p*K*_a_ of HBMQ^2+^ was measured using a photometric titration with BMQ^+^ as a base and Cl_3_CCOOH as acid. The equilibrium was monitored by ^1^H NMR. The resultant p*K*_a_ was calculated to be 9.1. This value is approximately one p*K*_a_ unit more acidic than HMQ^+^, an expected shift as making the compound a better electron acceptor has an inverse effect on proton accepting ability. By using the redox potentials of HBMQ^+^ and BMQ^+^ and the p*K*_a_ of HBMQ^2+^, the p*K*_a_ of the one-electron reduced species (HBMQ^+^) is calculated to be 17.7. This increase in p*K*_a_ upon reduction results in exergonic protonation of the reduced species by the ground-state of both [(dpab)_2_Ru(4,4′-dhbpy)]^2+^ and [(dpab)_2_Ru(4,4′-dhbpy)]^3+^ (Table 1).

In order for proton transfer and electron transfer reactions to be observed in the excited state, it is imperative that none of the reactions are exergonic in the ground state. Table 2 shows the free energies for reaction of both of the 4,4′-dhbpy complexes reported here with MQ^+^ and BMQ^+^ in CH_3_CN. It is clear that all ET, PT, and PCET reactions in the ground state are endergonic. Excited state electron transfer (ET*) is much less endergonic for both complexes with both acceptors, but is still energetically uphill. Excited state proton transfer (PT*) is also endergonic for both complexes with both acceptors, but by a smaller margin than PT. For PCET*, the change in redox potential with protonation shifts the MQ^+^ and BMQ^+^ reduction potentials to more positive values (see Figure S9 for BMQ^+^) resulting in exergonic PCET*.

**Table 2:** Free Energies for Ground and Excited State Reactions of bpy and dpab Complexes^a^

	ground-state reactions			excited-state reactions		
	Δ*G*_ET_ (kcal/mol)	Δ*G*_PT_ (kcal/mol)	Δ*G*_PCET_ (kcal/mol)	Δ*G*_ET*_ (kcal/mol)	Δ*G*_PT*_ (kcal/mol)	Δ*G*_PCET*_ (kcal/mol)
MQ^+^						
[(bpy)_2_Ru(4,4′-dhbpy)]^2+^	52	10.0	35	6.4	6.9	–9.3
[(dpab)_2_Ru(4,4′-dhbpy)]^2+^	55	7.4	40	13	2.8	–3.2
BMQ^+^						
[(bpy)_**2**_Ru(4,4′-dhbpy)]^**2+**^	50.8	11.7	33	4.4	8.5	–9.9
[(dpab)_2_Ru(4,4′-dhbpy)]^2+^	52.6	9.0	41	10	4.5	–2.4

a The approaches used for determination of the free energies are discussed on pages 17 and 18 of the SI.

Comparison of the free energies for the bpy and dpab complexes shows that the free energy for excited state electron transfer (ET*) is relatively more energetically uphill for the dpab complex, while the excited state proton transfer for [(dpab)_2_Ru(4,4′-dhbpy)]^2+^ is less endergonic with both MQ^+^ and BMQ^+^. In our previous report on [(bpy)_2_Ru(4,4′-dhbpy)]^2+^, it was observed that, despite the fact that the ET* free energy was around +6 kcal/mol for electron transfer to MQ^+^, transient absorption indicated that the electron transfer reaction still occurred. The change in the free energy for ET* from +6 to +13 kcal/mol upon substitution of dpab for bpy should have the effect of decreasing the rate constant for ET*, perhaps opening the door to observation of PCET*.

#### Possible Ground State H-Bond Formation

Upon changing the oxidation potential of the excited state by changing the spectator ligands (bpy to dpab), the acidity of the excited state is enhanced. Incorporating the amide functional groups on the 2,2′-bipyridine ring decreases the ground state p*K*_a_ to 14.4, which is nearly an order of magnitude more acidic than the corresponding [(bpy)_2_Ru(4,4′-dhbpy)]^2+^ excited state.

The role of acidity in proton-coupled electron transfer reactions is seen in the dependence of the reaction on H atom distance from the donor molecule to the acceptor. The inverse relationship between the rate of the PCET and the distance of the proton from the acceptor has been detailed in theoretical studies.^18^ With this distance dependence in mind, a key factor in enhancing the rate of proton transfer will be enhancing hydrogen bonding interactions between the chromophore and quencher. By increasing the equilibrium constant for full proton transfer, the equilibrium for hydrogen-bonding should also be enhanced.

While the hydroxy substituent of 4,4′-dhbpy in the bpy and dpab complexes is very weakly acidic, ground state H-bonding with pyridine was observed and measured by ^1^H NMR for the bpy complex in CD_3_CN.^1^ However, both MQ^+^ and BMQ^+^ are significantly weaker bases than pyridine, and spectroscopic methods as well as isothermal calorimetry failed to provide a measure of the H-bonding equilibrium constant for either of the 4,4′-dhbpy complexes. In spite of this, knowing that the acidity is correlated with hydrogen bond donor ability, it can be inferred that [(dpab)_2_Ru(4,4′-dhbpy)]^2+^ should be a better hydrogen bond donor than [(bpy)_2_Ru(4,4′-dhbpy)]^2+^.

### Experimental Approach to Differentiating ET*, PT*, and PCET* in These Systems

**Scheme 1:**
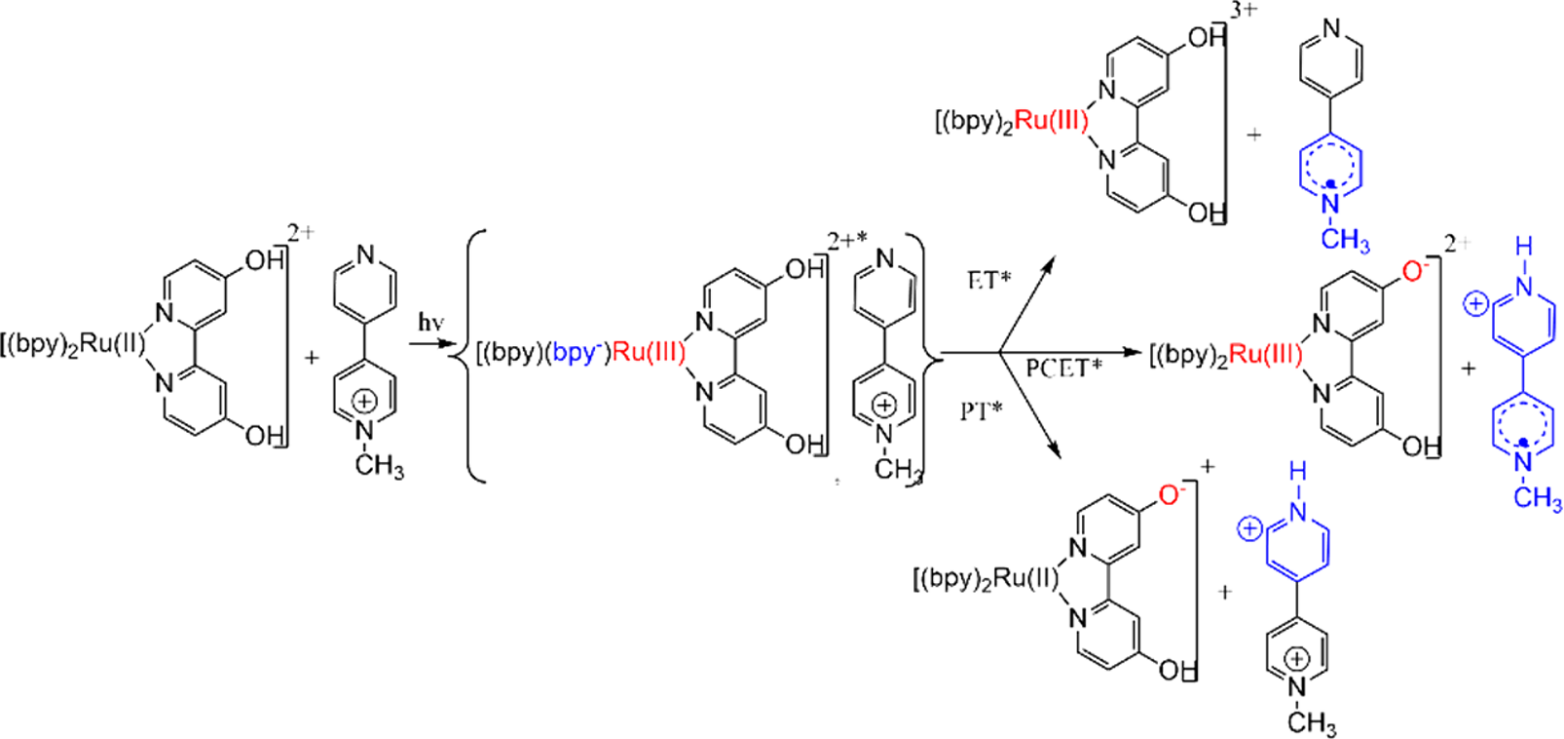
Formation and Reaction Products Emerging from the Encounter Complex for [(bpy)_2_Ru(4,4′-dhbpy)]^2+*^ Reaction with MQ^+^

Table 2 shows that the energetically favorable excited state reaction for each chromophore with each acceptor is PCET*. In previous work we clearly illustrated that, for the reaction of [(bpy)_2_Ru(4,4′-dhbpy)]^2+^ with MQ^+^, the products that emerged from the reaction cage following pulsed laser excitation were associated with ET*. Scheme 1 illustrates the reaction products possible for ET*, PT*, and PCET*. If a spectroscopic method can be found that *clearly* differentiates the possible cage escape products likely to form following pulsed laser excitation, then the primary reaction can be determined as long as possible following reactions are slow on the time scale of the spectroscopic method. For instance, in the specific case shown in Scheme 1, ET* can be followed by protonation of the MQ^0^ initially formed to yield HMQ^+^; the Ru^III^ complex formed initially would react with basic species in solution and the net change would be identical with the PCET* products. Fortunately, ns time-resolved transient absorption spectroscopy has the temporal resolution to directly observe the secondary reactions. The key, then, is determining in advance if the products of the three reaction paths are spectroscopically unique.

With the systems here, the visible (350–800 nm) spectra for the PT* reaction can be obtained from the difference of the spectrum of the protonated and deprotonated complexes. Neither the deprotonated or protonated forms of MQ^+^ (HMQ^2+^) and BMQ^+^ (HBMQ^2+^) absorb at all in the visible spectrum. The difference spectrum can be represented in the following way:
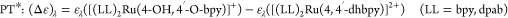
Both spectra are readily obtained.

Visible difference spectra for the ET* products require spectroelectrochemical (SEC) determination of the spectra for [(LL)Ru^III^(4,4′-dhbpy)]^3+^ (LL = bpy, dpab), MQ^0^, and BMQ^0^. Fortunately, all of these species are stable on the SEC time scale. The difference spectrum can be calculated from the starting complex and the SEC results (note: MQ^+^ and BMQ^+^ do not absorb in the visible spectrum):
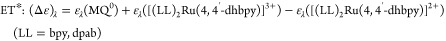


The spectra for the PCET* products are more difficult to definitively generate with the molecules in these reactions. Visible spectra are needed for the oxidized and deprotonated complexes; approaches to obtaining these are discussed below. In addition, the spectra of the reduced, protonated forms of the two quats are easily obtained. The difference spectrum is obtained from
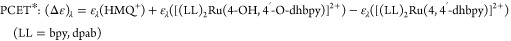


#### Generation of Predicted ET*, PT*, and PCET* Spectra for [(dpab)_2_Ru(4,4′-dhbpy)](PF_6_)_2_

The spectra required to generate simulated difference spectra for ET*, PT*, and PCET* were stated above. The UV–vis absorbance spectra for BMQ^+^ and HBMQ^2+^ were collected (Figure S8). The spectra show broad absorbance in the UV for both the BMQ^+^ and HBMQ^+^ with absorbance tailing out to 350 nm. These are therefore of no consequence for transient absorption spectral analysis between 350 and 800 nm. Upon reduction of BMQ^+^ by one-electron, new absorbance appears with a narrow transition having a maximum at 350 nm, and a second visible absorbance with a maximum at 535 nm (Figure S10). The absorbance features parallel what is seen with MQ^0^. The spectrum of the reduced and protonated species (HBMQ^+^) is similar to that of methyl viologen, with a narrow maximum around 400 nm and broad, visible absorbance with a maximum at 595 nm (Figure S10).

The chromophore, [(dpab)_2_Ru(4,4′-dhbpy)]^2+^, shows strong absorbance in the UV attributed to the ligand localized π to π* transitions. The metal-to-ligand charge transfer (MLCT) transition characteristic of ruthenium diimine complexes is red-shifted compared to [(bpy)_2_Ru(4,4′-dhbpy)]^2+^ (490 nm). Upon deprotonation with one equivalent of a strong base, the MLCT shifts to lower energy (520 nm, Figure S5). These two spectra were used to generate the simulated PT* spectrum.

**Figure 3:**
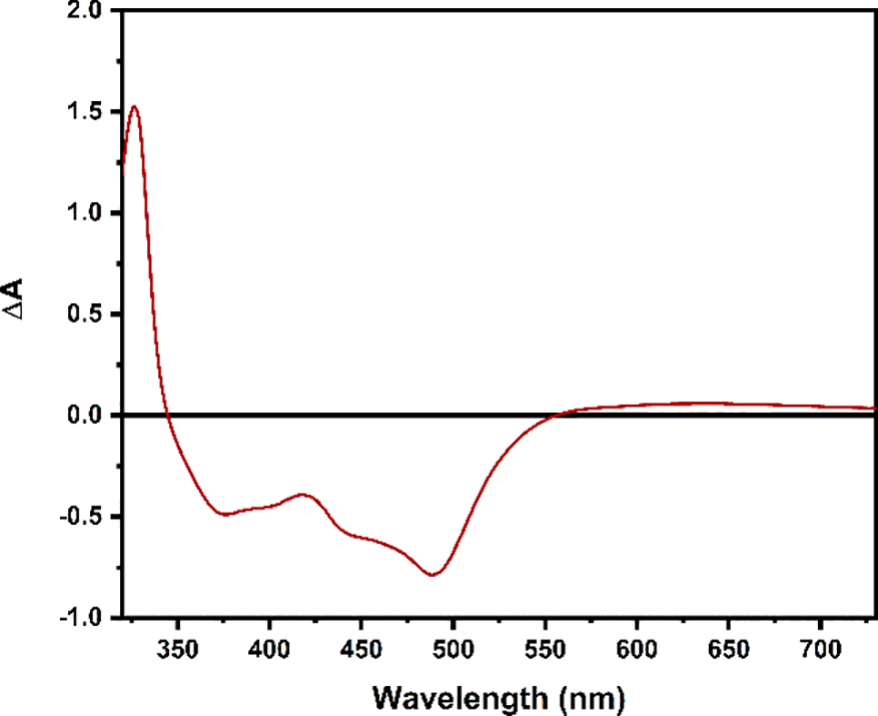
Difference absorption spectrum for [(dpab)_2_Ru(4,4′-dhbpy)]^3+^ generated by spectroelectrochemistry in acetonitrile.

Spectroelectrochemistry was used to obtain the absorbance spectrum of the one-electron oxidized complex. Figure 3 shows the difference absorption spectrum of this species, with a characteristic bleach of the visible MLCT absorbance seen in Ru^II^ compounds of this nature and only very weak absorption features in the visible spectrum (λ > 350 nm) for the Ru^III^ complex.

**Figure 4:**
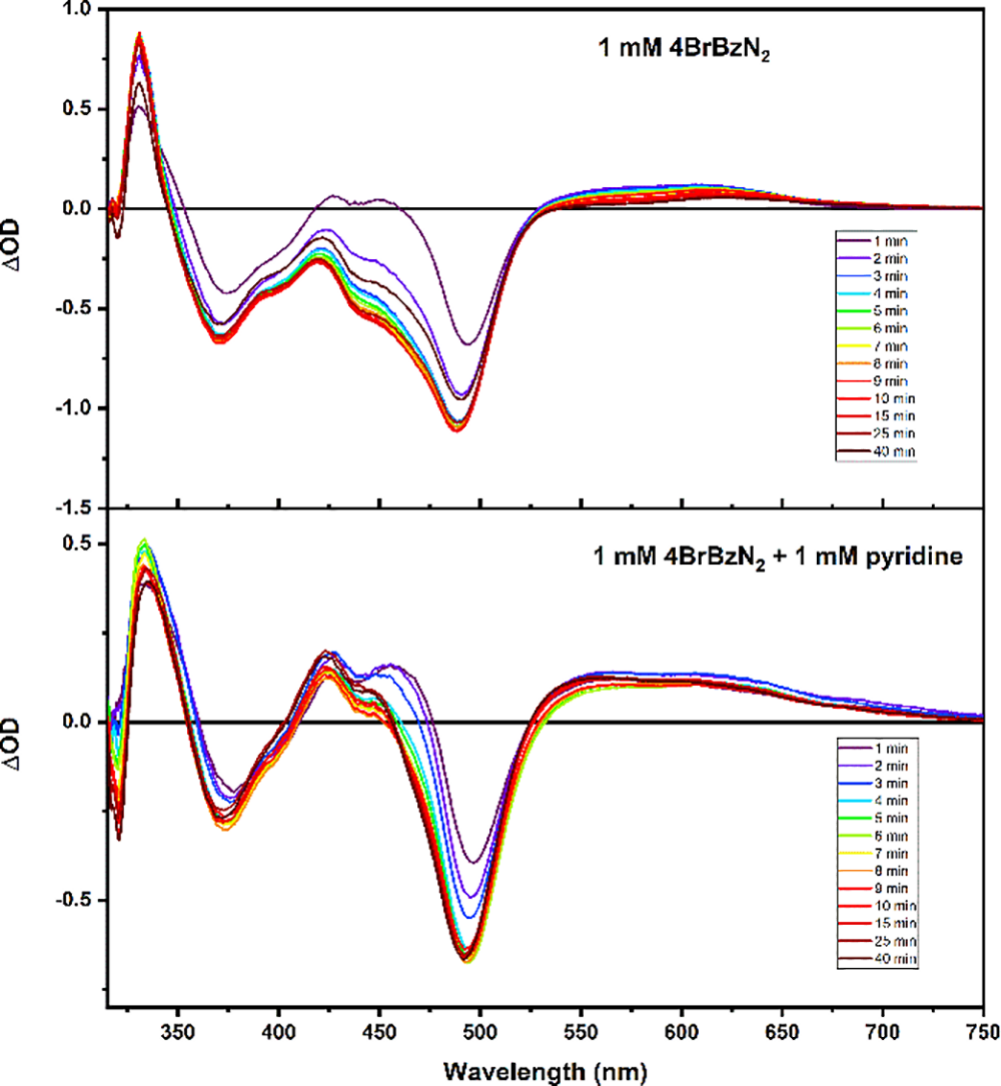
Difference absorption spectra of [(dpab)_2_Ru(4,4′-dhbpy)]^3+^ (top) and the monodeprotonated complex, [(dpab)_2_Ru(4-(OH)-4′-(O)-bpy)]^2+^ (bottom).

Generation of the spectrum of the one-electron oxidized, deprotonated species ([(LL)_2_Ru(4-OH,4′-O-dhbpy)]^2+^) was accomplished by an irreversible photoinduced electron transfer reaction in the presence of base since spectroelectrochemistry in basic solution failed to provide a viable spectrum. Here, the irreversible oxidative quencher, 4-bromobenzene diazonium (BrBzN_2_) tetrafluoroborate (*E*_p,c_ = −0.4 V vs Fc^+/0^) was used, along with pyridine as a base.^19^ Neither pyridine, pyridinium, BrBzN_2_ and its decomposition products absorb in the visible region of the spectrum.^20^ Steady-state photolysis was conducted until the reaction reached an end point, signified by a lack of change in the absorbance spectrum with further irradiation. The spectrum is shown as a difference spectrum in Figure 4; also included is the photochemically generated difference spectrum in the absence of pyridine, which is identical with the spectrum obtained from the one-electron oxidized complex by spectroelectrochemistry (Figure 3). The most significant differences between the spectra are seen in positive Δ*A* values around 425 and 550 nm, where there is much greater absorbance for the deprotonated, oxidized complex than just the oxidized complex itself. These absorbance features can aid in distinguishing between the two species in transient absorption experiments.

**Figure 5:**
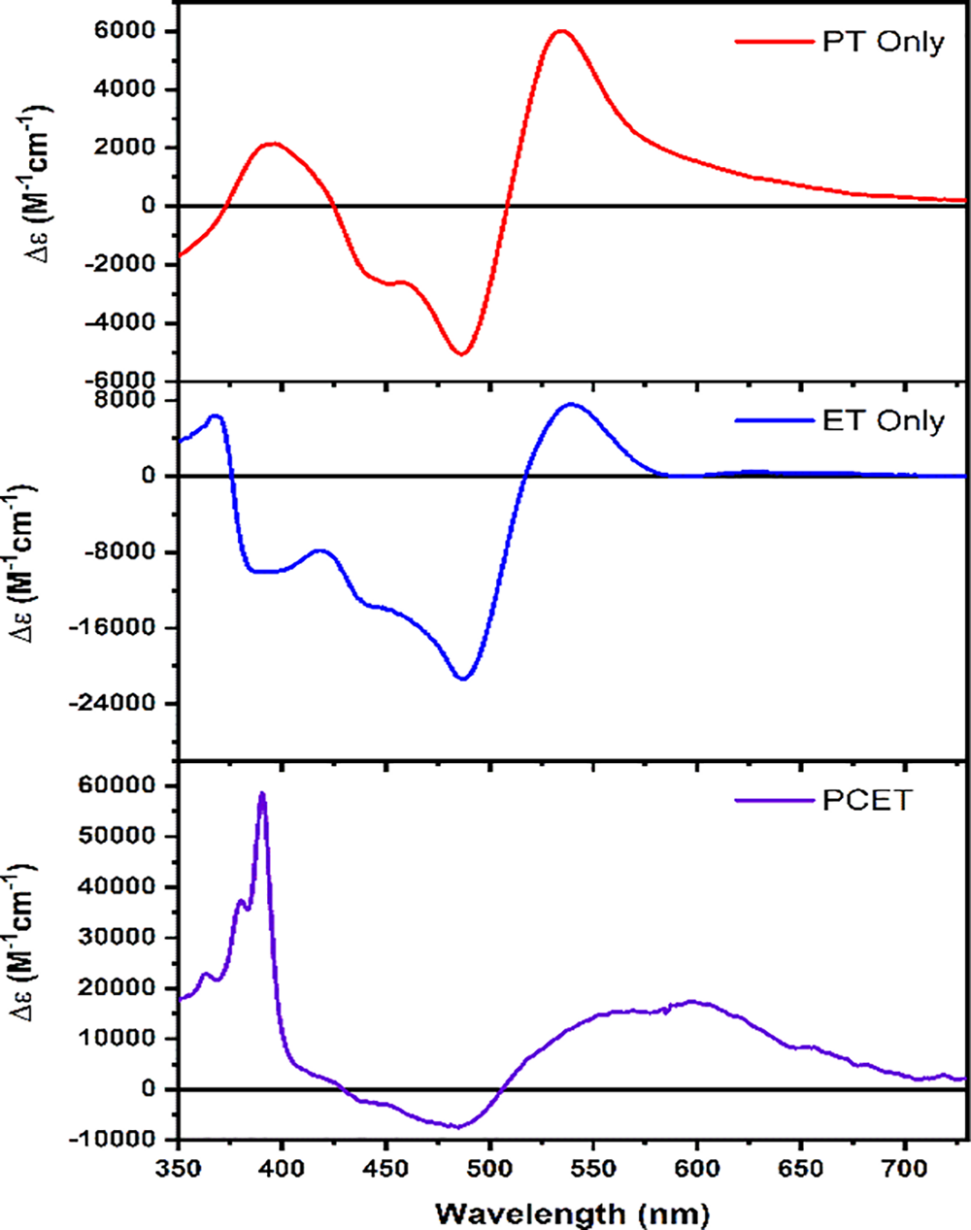
Spectra of PT* (top), ET* (middle), and PCET* (bottom) products for reactions between [(dpab)_2_Ru(4,4′-dhbpy)]^2+^ and MQ^+^.

Combining the spectra of the different oxidation/protonation states of the chromophore, [(dpab)_2_Ru(4,4′-dhbpy)]^2+^, and the quenchers, MQ^+^ and BMQ^+^, yields the concomitant difference spectra for the various possible products of the photochemical reaction between the chromophore and quencher. The ET* only, PT* only, and PCET* difference spectra are shown in Figure 5. Values of Δε at each wavelength were determined as stated previously.

It is clear from the calculated spectra that distinct differences exist between each of the three potential products and that identification of the cage escape products for a reaction between the dpab complex chromophore and MQ^+^ should be straightforward. The same is true for reaction of the photoexcited dpab complex and BMQ^+^.

### Excited State Quenching and Nanosecond Transient Absorption of Complexes with MQ^+^ and BMQ^+^

Armed with the expected difference spectra for the ET*, PT*, and PCET* products, nanosecond transient absorption was used to observe the reaction between the excited states of [(dpab)_2_Ru(4,4′-dhbpy)]^2+^ and [(bpy)_2_Ru(4,4′-dhbpy)]^2+^ and the e^–^/H^+^ acceptors MQ^+^ and BMQ^+^, revealing the products escaping the reaction cage. The photoinduced reaction between [(bpy)_2_Ru(4,4′-dhbpy)]^2+^ and MQ^+^ was previously reported.^11^

#### [(bpy)_2_Ru(4,4′-dhbpy)]^2+^ Quenching with BMQ^+^

**Figure 6:**
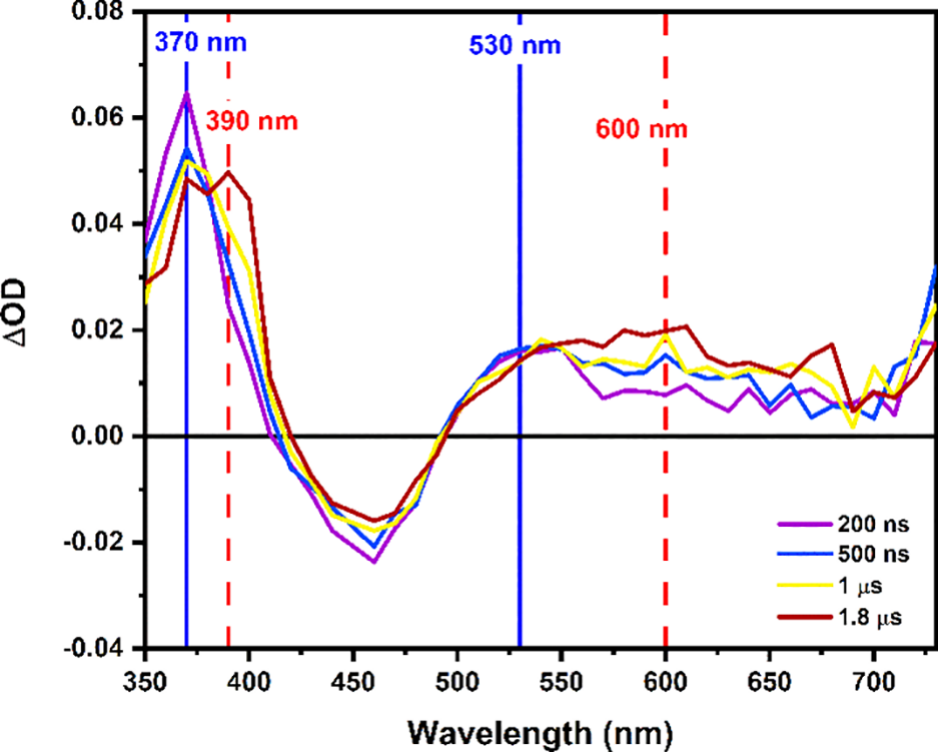
Transient absorption spectrum of [(bpy)_2_Ru(4,4′-dhbpy)]^2+^ in the presence of 200 mM BMQ^+^ in acetonitrile shown at 200 ns, 500 ns, 1 μs, and 1.8 μs after the laser pulse.

Quenching with BMQ^+^ is reminiscent of previous studies with MQ^+^ where ET* was followed by PT.^11^ Excited state quenching followed Stern–Volmer kinetics and resulted in a rate constant of 2.9 × 10^8^ M^–1^ s^–1^. The resulting transient absorption spectrum is shown in Figure 6 over the first 2 μs of reaction between BMQ^+^ and [(bpy)_2_Ru(4,4′-dhbpy)]^2+^. After 200 ns, the initial reaction products are seen with absorbance maxima at 370 and 530 nm. This is consistent with absorption of BMQ^0^ (Figure S10). Over the span of 1 μs the final reaction products are generated with absorbance maxima at 390 and 600 nm, indicative of HBMQ^+^ absorption. There is also a contribution to the absorbance at around 520 nm from the oxidized, deprotonated ruthenium complex (note: the simulated transient difference spectra differ from those of Figure 5, but parallel those of ref (11)). This case is analogous to the reaction with MQ^+^, and as a result, the rate constant provided above is a reflection of the rate constant for excited-state quenching via electron transfer. The subsequent protonation of BMQ^0^ occurs at diffusion limited rates, and the rate constant was extracted from global analysis of the transient absorption data. It can be speculated that, like with the MQ^+^ system, the protonation of BMQ^0^ occurs by proton exchange with both the Ru^III^ that was generated in the photoreaction and the bulk Ru^II^ in solution. Control studies with [(bpy)_2_Ru((OMe)_2_-bpy)]^2+^ ((OMe)_2_-bpy = 4,4′-dimethoxy-2,2′-bipyridine) were not conducted, as the resulting spectra and previously reported studies clearly inform the analysis of the transient absorption studies. The bottom line is that the behavior of this system is ET*/PT, just like reaction of [(bpy)_2_Ru(4,4′-dhbpy)]^2+^ with MQ^+^.

#### [(dpab)_2_Ru(bpy)]^2+^ and MQ^+^

In this case we decided to examine possible ET* reaction of the control complex lacking hydroxyl substituents, [(dpab)_2_Ru(bpy)]^2+^, with MQ^+^. No change in the excited-state lifetime of [(dpab)_2_Ru(bpy)]^2+*^ was observed with added MQ^+^ up to its solubility limit indicating that *no oxidative quenching* of the excited state occurs. The same observation is made when the more easily reduced BMQ^+^ is used as the quencher. The resulting spectra and Stern–Volmer plots are found in Figures S11 and S12. The implication is that ET* reaction of MQ^+^ and BMQ^+^ with the photoexcited complex having hydroxylated bpy ligands, [(dpab)_2_Ru(4,4′-dhbpy)]_2_]^2+^, is unlikely. The Ru^III/II^ potential for the control complex ([(dpab)_2_Ru(bpy)]^2+^) was measured by cyclic voltammetry as 1.01 V vs Fc^+/0^ and the emission energy was 1.95 eV. The free energy for ET* can be calculated from the *E*^0^(III/II*) of −0.94 V vs Fc^+/0^ and the MQ^+/0^ potential of −1.35 V vs Fc^+/0^ to be approximately +9 kcal/mol (−0.41 V). This is *less* endergonic than the oxidative quenching of [(dpab)_2_Ru(4,4′-dhbpy)]^2+^ by MQ^+^ (Table 2). This further supports the contention that ET* between [(dpab)_2_Ru(4,4′-dhbpy)]^2+^ and MQ^+^ is not likely to occur.

Despite the result of the control reaction and the endergonicity of the ET* reaction of [(dpab)_2_Ru(4,4′-dhbpy)]^2+^ with MQ^+^, the observed quenching reaction between the two, discussed below, indicates that either PT* or PCET* is occurring. The free energy for the PCET* reaction is −3.2 kcal/mol (Table 2). Although this is less exergonic than that for [(bpy)_2_Ru(4,4′-dhbpy)]^2+^, which followed an ET*/PT path, the dpab system remains amenable to PCET*.

#### Photoreaction of [(dpab)_2_Ru(4,4′-dhbpy)]^2+^ with MQ^+^ and BMQ^+^

Quenching rate constants are shown in Table 3 for reaction of the bpy and 4,4′-dhbpy complexes with MQ^+^, BMQ^+^, and a base, 3-acetylpyridine, with a p*K* value similar to that of MQ^+^.

**Table 3:** Rate Constants Taken from Stern–Volmer Analysis of Kinetic Data in Acetonitrile^a^

	BMQ^**+**^	MQ^**+**^	3-acetylpyridine
[(bpy)_2_Ru(4,4′-dhbpy)]^2+^	2.9 × 10^8^ M^–1^ s^–1^	6.7 × 10^7^ M^–1^ s^–1^ ^11^	–
[(dpab)_2_Ru(4,4′-dhbpy)]^2+^	1.6 × 10^7^ M^–1^ s^–1^	1.1 × 10^7^ M^–1^ s^–1^	1.3 × 10^7^ M^–1^ s^–1^

a For quenching rate constants with BMQ^+^ and MQ^+^ listed in the table, the total ion concentration of the solution was kept constant at 200 mM by addition of tetrabutyl ammonium hexafluorophosphate.

Rate constants for quenching of the excited-state of [(dpab)_2_Ru(bpy)]^2+^ were omitted from the table, as no measurable quenching occurred between the bipyridinium quenchers and the complex.

**Figure 7:**
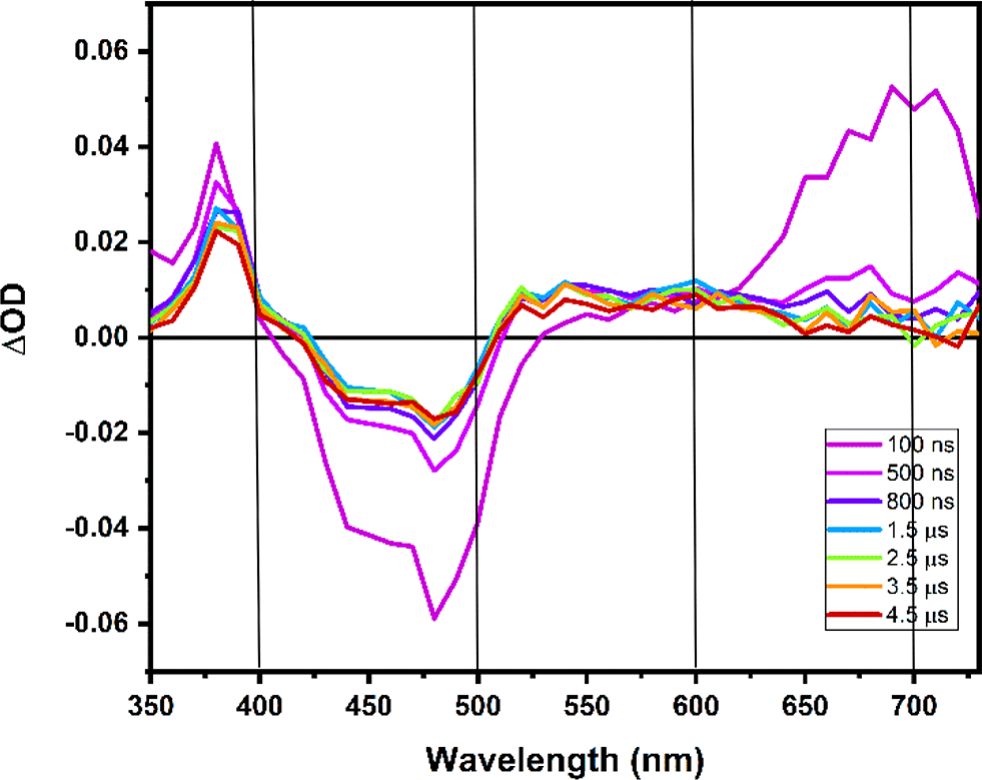
Transient absorption spectrum of [(dpab)_2_Ru(4,4′-dhbpy)]^2+^ in the presence of 140 mM MQ^+^ and 60 mM TBAPF_6_ in acetonitrile. λ_ex_ = 450 nm.

**Figure 8:**
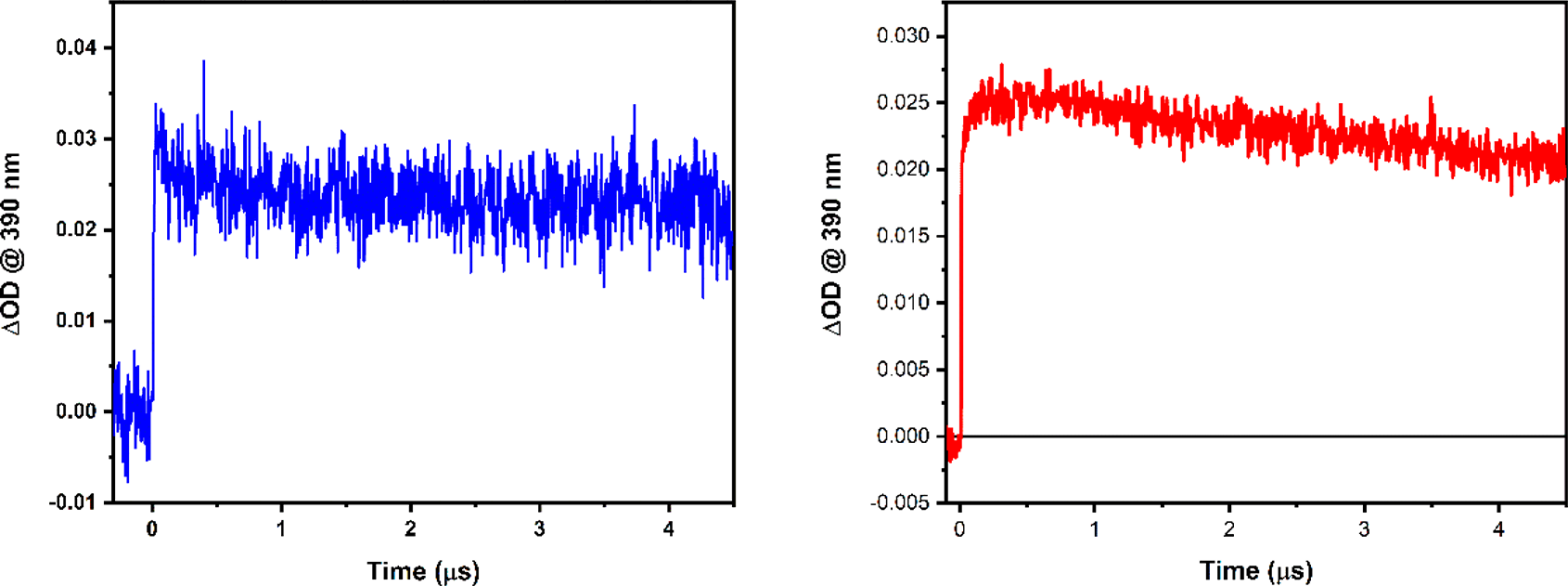
Kinetics at 390 nm following 450 nm excitation for reaction of [(dpab)_2_Ru(4,4′-dhbpy)]^2+^ with (left) 500 mM 3-acetylpyridine and (right) 140 mM MQ^+^ with 60 mM TBAPF_6_ in acetonitrile.

The transient absorption spectrum for reaction of photoexcited [(dpab)_2_Ru(4,4′-dhbpy)]^2+^ with MQ^+^ and the 390 nm kinetic decay are shown in Figures 7 and 8, respectively. Proton transfer and proton-coupled electron transfer products yield distinct absorbance features throughout the visible spectrum as shown in the simulated spectra of Figure 5. The resulting TA spectrum for the photoinduced reaction between [(dpab)_2_Ru(4,4′-dhbpy)]^2+^ and MQ^+^, Figure 7, shows that, at early times, <100 ns, the excited-state absorbance features are observed (see Figure S13 for spectrum of [(dpab)_2_Ru(4,4′-dhbpy)]^2+*^, ΔOD values above 650 nm reflect ineffective correction for emission; see Experimental Section), followed by a growth in absorbance below 400 nm and between 500 and 650 nm (lacking any clear maximum). What is clear from the transient absorption data is that there is no distinct peak at 530 nm indicating PT* has occurred as the predominant, or preliminary photoproduct. Rather, the absorbance features in this spectrum more closely parallel what is expected for the concerted proton and electron transfer products (see Figure 5).

In addition, the kinetics at 390 nm can be compared for the PT* reaction with a base (3-acetylpyridine, see Figure S14) and the observed reaction with MQ^+^. Figure 8 shows the 390 nm kinetics observed following excitation at 450 nm for excited state reaction with the base and MQ^+^. Had the process involved ET*, a net bleach would have been expected after the relaxation of the excited state absorption at 390 nm (spectrum shown in Figure 5). For the PT* reaction with 3-acetylpyridine, net absorption is observed following decay of the more strongly absorbing excited state.

In the presence of MQ^+^ an absorbance rise at 390 nm over the first 300 ns is observed (Figure 8). This, coupled with the strong resemblance of the overall observed spectrum with the simulated PCET* rather than the PT* spectrum, can be taken as spectroscopic evidence for PCET* between [(dpab)_2_Ru(4,4′-dhbpy)]^2+^ and MQ^+^. In addition, the thermodynamic favorability of PCET* over PT* and ET* augments the evidence to suggest that the mechanism by which excited-state deactivation occurs is via concerted proton and electron transfer in the excited state encounter complex.

One thing not shown in Scheme 1 is that the PT* reaction actually yields the deprotonated Ru complex in an electronically excited state. Hypothetically, this excited complex could then react via electron transfer with MQ^+^ or HMQ^2+^. However, the deprotonated complex has a very short (<5 ns Table 1) lifetime, so bimolecular quenching by HMQ^2+^ is not possible due to the low concentration of HMQ^2+^ formed via PT*. As for possible reaction of the deprotonated excited state (*E*^0 2+/+*^ = −1.08 V vs Fc^+/0^) with MQ^+^ (*E*^0 2+/+^ = −1.2 V vs Fc^+/0^), the reaction is endergonic and the quenching reaction is likely to have a quenching rate constant well below the diffusion limit. Even if this happened, it would lead to the formation of MQ^0^ and would appear spectroscopically like an ET*/PT reaction. The TA spectrum shows no evidence for ET* products.

**Figure 9:**
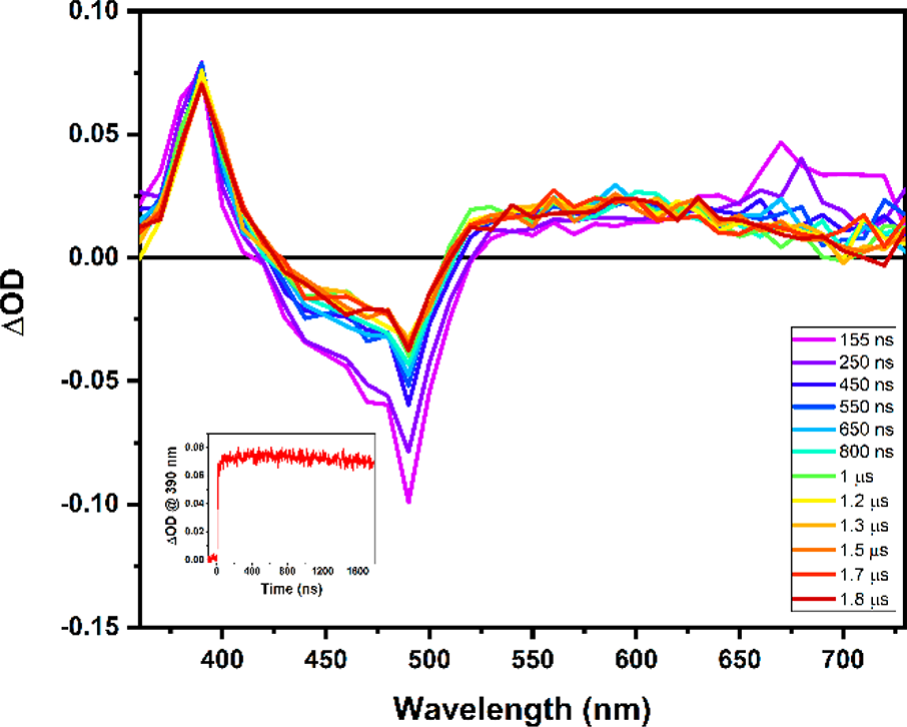
Transient absorption spectrum following 450 nm excitation of [(dpab)_2_Ru(4,4′-dhbpy)]^2+^ in the presence of 150 mM BMQ^+^ and 50 mM TBAPF_6_ in acetonitrile. The inset shows the single wavelength kinetics at 390 nm.

Figure 9 shows the transient spectrum for the reaction of BMQ^+^ with the excited-state of [(dpab)_2_Ru(4,4′-dhbpy)]^2+^. Excited state proton transfer is slightly less favorable and ET* is slightly less endergonic for the reaction of excited [(dpab)_2_Ru(4,4′-dhbpy)]^2+^ and BMQ^+^. Despite this, ET* does not appear to occur, as demonstrated by the lack of excited-state quenching of [(dpab)_2_Ru(bpy)]^2+^ by BMQ^+^ and the TA spectrum of the cage escape products (Figure 9) that are very similar to the products generated by quenching of [(dpab)_2_Ru(4,4′-dhbpy)]^2+^* by MQ^+^. Thus, the photoreactions of [(dpab)_2_Ru(4,4′-dhbpy)]^2+^ with MQ^+^ and BMQ^+^ are the same.

#### Kinetic Deuterium Isotope Effects for the Reaction of [(dpab)_2_Ru(4,4′-dhbpy)]^2+^ with MQ^+^ and BMQ^+^

Kinetic deuterium isotope effects for the excited-state reactions between [(dpab)_2_Ru(4,4′-dhbpy)]^2+^ and the bipyridinium quenchers were probed. In order to measure the *k*_H_/*k*_D_ values, the deuterated complex was prepared, and transient absorption experiments were conducted in deuterated acetonitrile. Table 4 shows the observed *k*_H_/*k*_D_ for the [(dpab)_2_Ru(4,4′-dhbpy)]^2+^ as well as [(bpy)_2_Ru(4,4′-dhbpy)]^2+^ photoreactions with MQ^+^ and BMQ^+^. No substantial deuterium kinetic isotope effect was measured by way of Stern–Volmer quenching. It is possible that the lack of KIE can be explained by the fact that there is only a small degree of excited-state quenching for the reaction between [(dpab)_2_Ru(4,4′-dhbpy)]^2+^ and the respective quenchers. Given the small absolute difference in excited-state lifetime that was measured, the error in such rate constant measurements can be obscured by the margin of error in the excited state decays, making it difficult to observe *k*_H_/*k*_D_ values that are close to unity. Recently Hammarstrom et al. have noted that deuterium isotope effects vary over a wide margin for PCET reactions and the absence of a large isotope effect does not necessarily exclude PCET as a reaction path.^22^

**Table 4:** Kinetic Isotope Effects for Excited State Quenching of Each Complex by MQ+ and BMQ+

	*k*_H_/*k*_D_ MQ^+^	*k*_H_/*k*_D_ BMQ^+^
[(dpab)_2_Ru(4,4′-dhbpy)]^2+^	0.92	1.30
[(bpy)_2_Ru(4,4′-dhbpy)]^2+^	0.90^11^	1.04

## Conclusions

This work demonstrates how manipulation of the thermochemical properties of a transition metal chromophore can be used to exert control over excited-state reactivity in systems where ET*, PT*, or PCET* can occur. Comparing [(dpab)_2_Ru(4,4′-dhbpy)]^2+^ and [(bpy)_2_Ru(4,4′-dhbpy)]^2+^, it is clear that changing the excited-state p*K*_a_ along with the Ru^III/II^ potential through incorporating electron withdrawing amide functional groups on the spectator ligands is sufficient to switch the mechanism from ET* to PCET* for quenching reactions with bipyridinium salts. This further demonstrates the role of acidity in dictating PCET reactivity.

Transient absorption spectroscopic data supported that a concerted PCET* pathway for the reaction of the MLCT triplet state of [(dpab)_2_Ru(4,4′-dhbpy)]^2+^ with both MQ^+^ and BMQ^+^. PT* reactivity of these systems was possible thermodynamically, but the distinct spectroscopic features associated with the proton transfer products were not observed. In addition, with BMQ^+^, there appears to be a modest kinetic isotope effect, furthering the case for PCET*. Also explored was an expansion of the study of [(bpy)_2_Ru(4,4′-dhbpy)]^2+^ by investigating the excited-state quenching by BMQ^+^. Transient spectroscopic analysis showed that excited-state quenching occurs via an ET*/PT pathway, much like in the previous study with MQ^+^.^11^ Future work will develop the ideas further by exploring the free energy region over which ET*, PT*, and PCET* are observed in closely related complexes.

## Supporting Information Available

^1^H and COSY NMR spectra of the dpab complex, cyclic voltammetric and spectroelectrochemical results for the dpab complexes and BMQ^+^, absorption spectra of the dpab complexes, BMQ^+^ and HBMQ^2+^, data for determination of the p*K*_a_ for [(dpab)_2_Ru(4,4′-dhbpy)]^2+^, quenching data for [(dpab)_2_Ru(bpy)]^2+^ with MQ^+^, BMQ^+^, and acetylpyridine, a table of thermochemical data for ground state reactions of chromophores with complexes, and a detailed explanation of the determination of the free energies for PT, ET, PCET, PT*, ET*, and PCET* (PDF)
