# Supplementary material for: Bimolecular Excited-State Proton-Coupled Electron Transfer within Encounter Complexes
Source: J Am Chem Soc. 2023 Feb 20;145(8):4462–72. doi: 10.1021/jacs.2c10165 (PMC9983007; doi:10.1021/jacs.2c10165)
Supplement: Supplementary file 1 — ja2c10165_si_001.pdf [file ja2c10165_si_001.pdf]

Bimolecular Excited-State Proton-Coupled Electron Transfer within Encounter Complexes

Kristina Martinez[a], Sydney M. Koehne[a] , Kaitlyn Benson[b] , Jared J. Paul[b*], Russell H. Schmehl[a*]

a. Department of Chemistry, Tulane University, New Orleans, LA, 70118, USA

b. Department of Chemistry, Villanova University, Philadelphia, PA, 19085, USA

c. Department of Chemistry, Northwestern U., Evanston, IL, 60208, USA

d. Division of Chemistry and Chemical Engineering, Caltech, Pasadena, CA, 91125, USA

Email: russ@tulane.edu

Supplementary Information

Fig. S1: NMR spectrum of $[(dpab)_2Ru(4,4'\text{-dhbpy})](PF_6)_2$ in CD_3CN (dpab= 4,4'-di(n-propyl)amido-2,2'-bipyridine, 4,4'-dhbpy= 4,4'-dihydroxy-2,2'-bipyridine. 2

Fig. S2: ^{1}H COSY spectrum of $[(dpab)_2Ru(bpy)](PF_6)_2$ in CD_3CN 3

Fig. S3: Oxidative and reductive CV of $[(dpab)_2Ru(4,4'\text{-dhbpy})](PF_6)_2$ in CD_3CN 4

Fig. S4: Oxidative and reductive CV of $[(dpab)_2Ru(4\text{-O-}4'\text{-OHbpy})](PF_6)_2$ in CD_3CN 5

Fig. S5: Absorption and Luminescence spectra of $[(dpab)_2Ru(4,4'\text{-dhbpy})]^{2+}$, $[(dpab)_2Ru(4\text{-O-}4'\text{-OHbpy})]^{+}$ and $[(dpab)_2Ru(4,4'\text{-Obpy})]^{0}$ in CD_3CN 6

Fig. S6: Steady-state UV-Vis titration with 2-aminobenzimidazole and (b) linear fit of concentration data derived from absorbance changes to determine the pKa of [Ru(dpab)2(4,4'-dhbpy)]. 7

Fig. S7: Absorption spectra of $[(dpab)_2Ru(4,4'\text{-dhbpy})]^{2+}$ in CH_3CN in the presence of (a) 1 mM 4-bromo-phenyldiazonium ($BrBzN_2$) and (b) 1 mM $BrBzN_2$ and 1 mM pyridine 8

Fig. S8: Absorption of BMQ^+ and $HBMQ^{2+}$ in CH_3CN 9

Fig. S9: CV of BMQ^+ in CH_3CN and CV of BMQ^+ + 0.02 M triflic acid in CH_3CN 10

Fig. S10: UV-vis spectra of $BMQ^+$and $HBMQ^{2+}$ upon one electron reduction in CH_3CN 11

Fig. S11, S12: Quenching of luminescence of $[(dpab)_2Ru(bpy)]^{2+}$ in CH_3CN by MQ^+ and BQ^+ 12

Fig. S13: nsTA spectra of $[Ru(dpab)_2(4,4'\text{-dhbpy})]^{2+}$in the absence and in the presence of MQ^+ 13

Table S1. Calculated Gibbs free energy for ground state reactions given in kcal/mol 14

Fig. S14: Description of PT* reaction of $[Ru(dpab)_2(4,4'\text{-dhbpy})]^{2+}$ and 3-acetylpyridine, TA 15

Equations used for Determination of Free Energies for ET*, PT* and PCET* 17

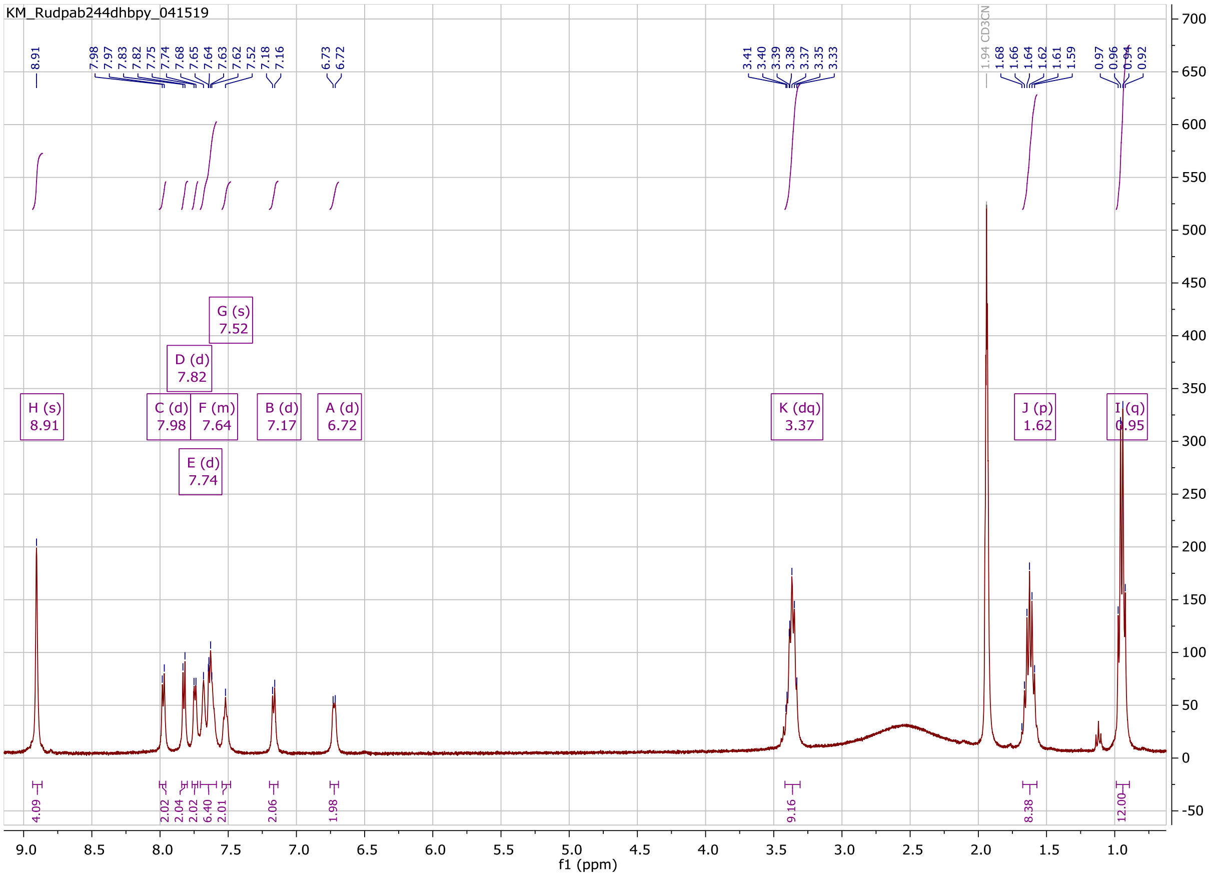

Figure S1. NMR spectrum of [(dpab)$_2$Ru(4,4'-dhbpy)](PF$_6$) in CD$_3$CN (dpab= 4,4'-di(n-propyl)amido-2,2'-bipyridine, 4,4'-dhbpy= 4,4'-dihydroxy-2,2'-bipyridine.

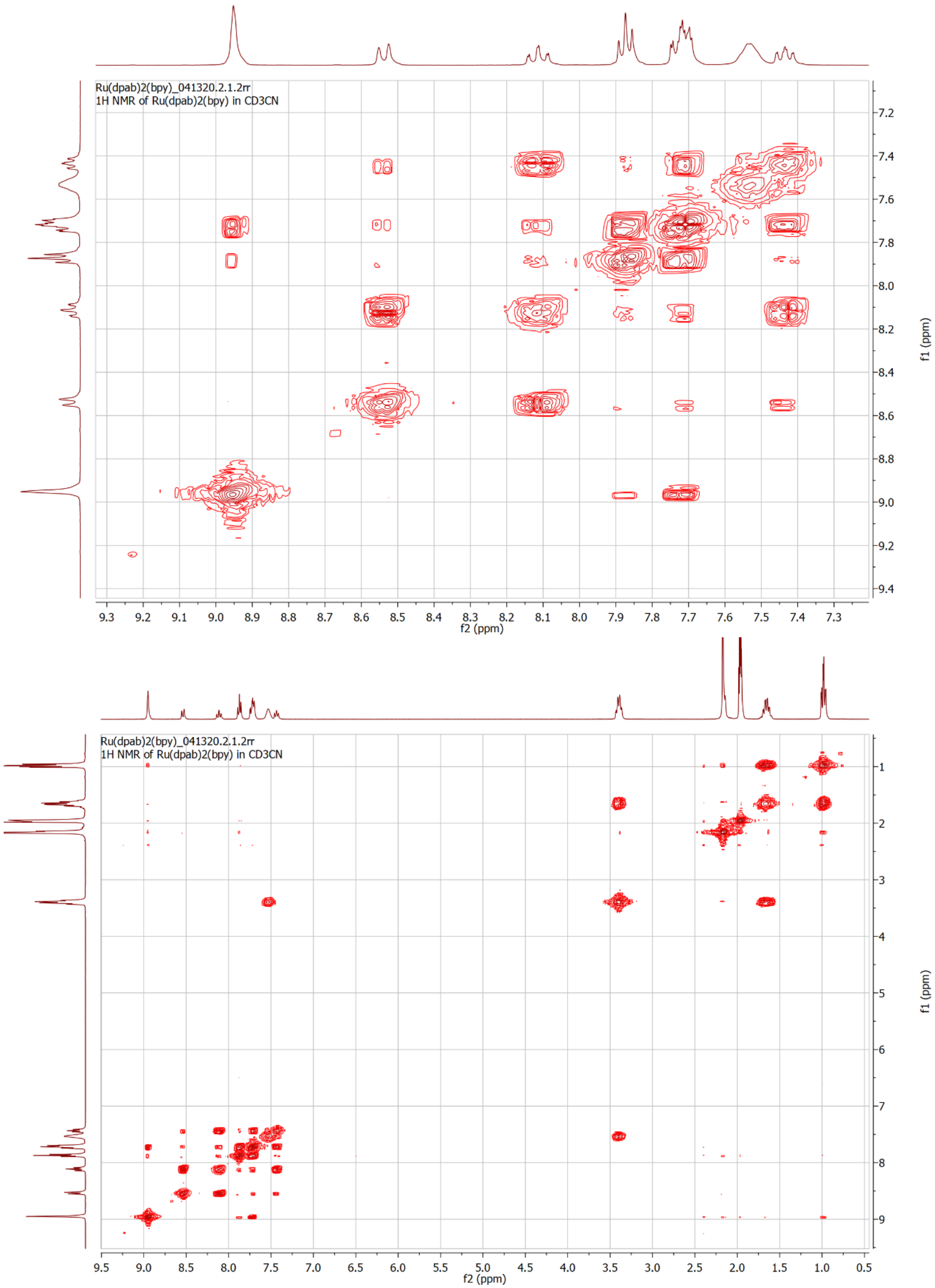

Figure S2. ^{1}H COSY spectrum of $[(dpab)_2Ru(bpy)](PF_6)_2$ in CD_3CN

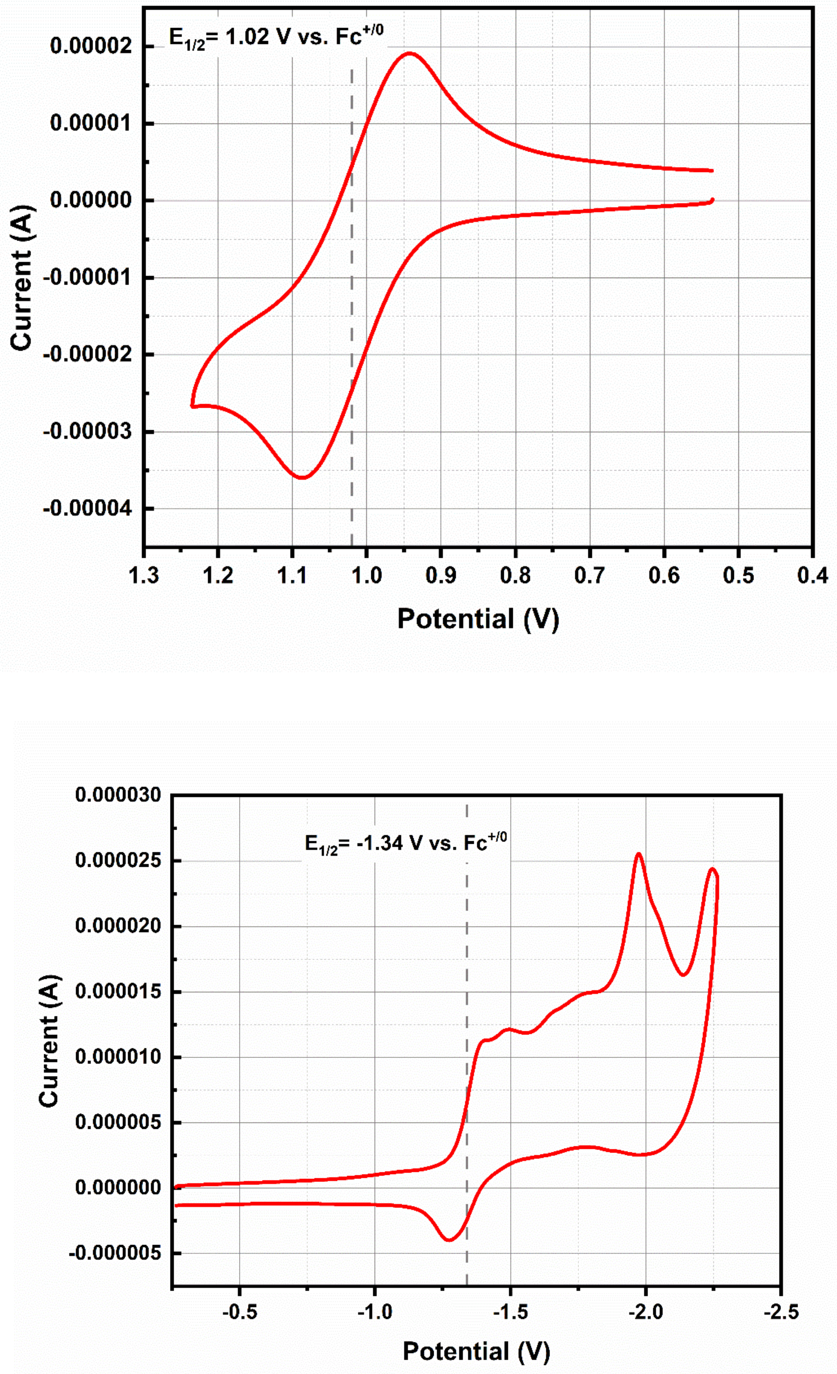

Figure S3. Oxidative and reductive CV of [(dpab)$_2$Ru(4,4'-dhbpy)](PF$_6$)$_2$ in CD$_3$CN

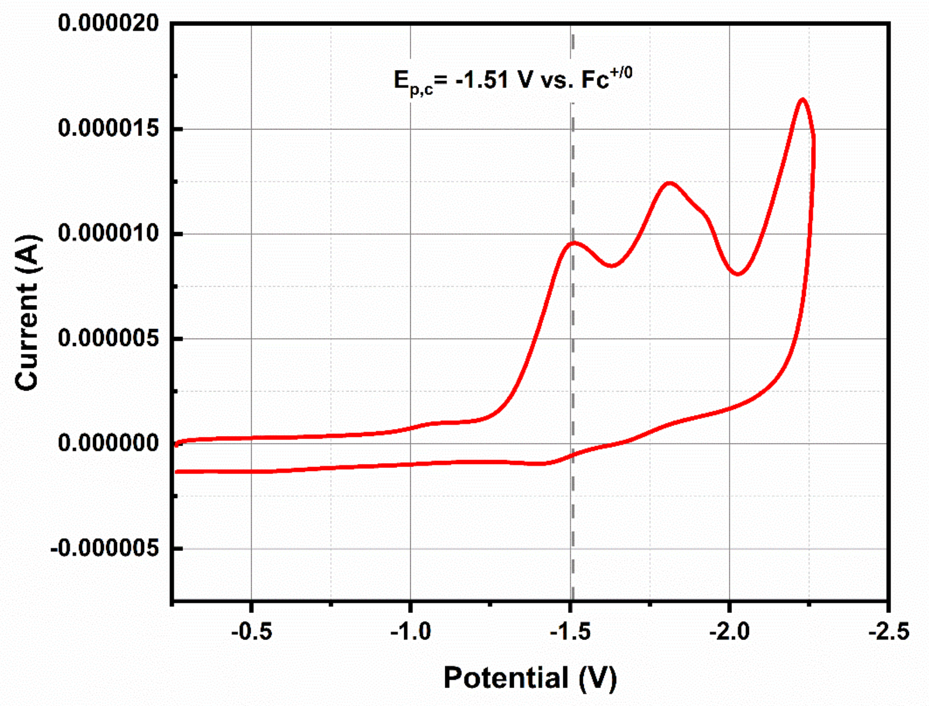

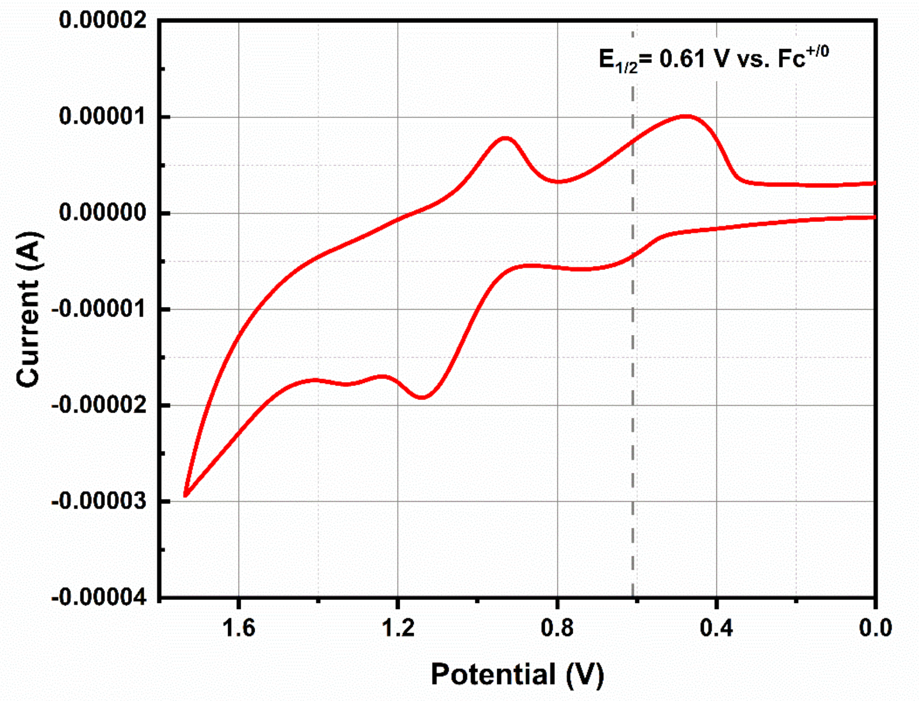

Figure S4. Oxidative and reductive CV of [(dpab)$_2$Ru(4-O-4'-OHbpy)](PF$_6$)$_2$ in CD$_3$CN

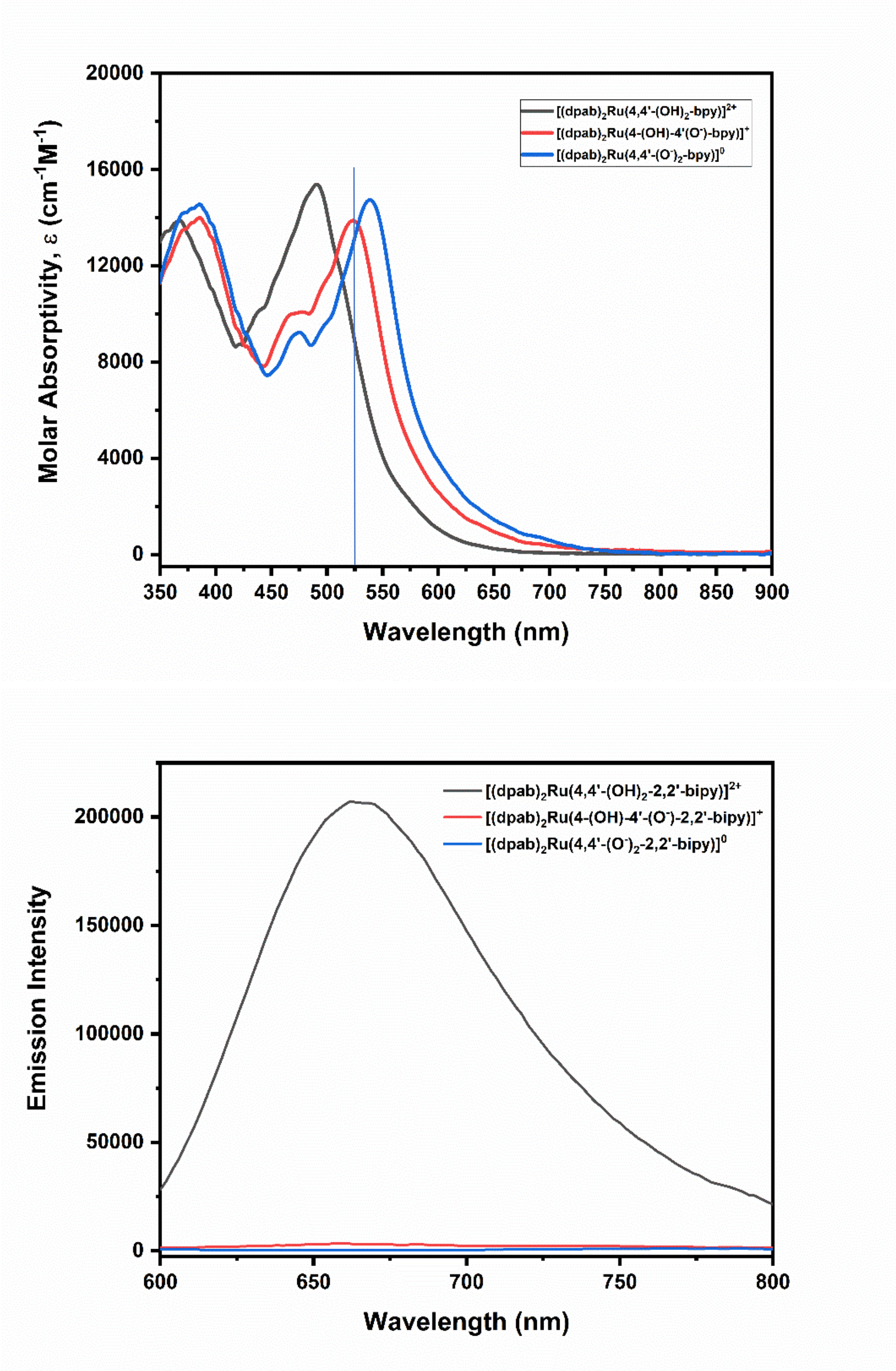

Figure S5. Absorption and Luminescence spectra of $[(dpab)_2Ru(4,4'\text{-}dhbpy)]^{2+}$, $[(dpab)_2Ru(4\text{-}O\text{-}4'\text{-}OHbpy)]^{+}$ and $[(dpab)_2Ru(4,4'\text{-}Obpy)]^{0}$ in CD_3CN

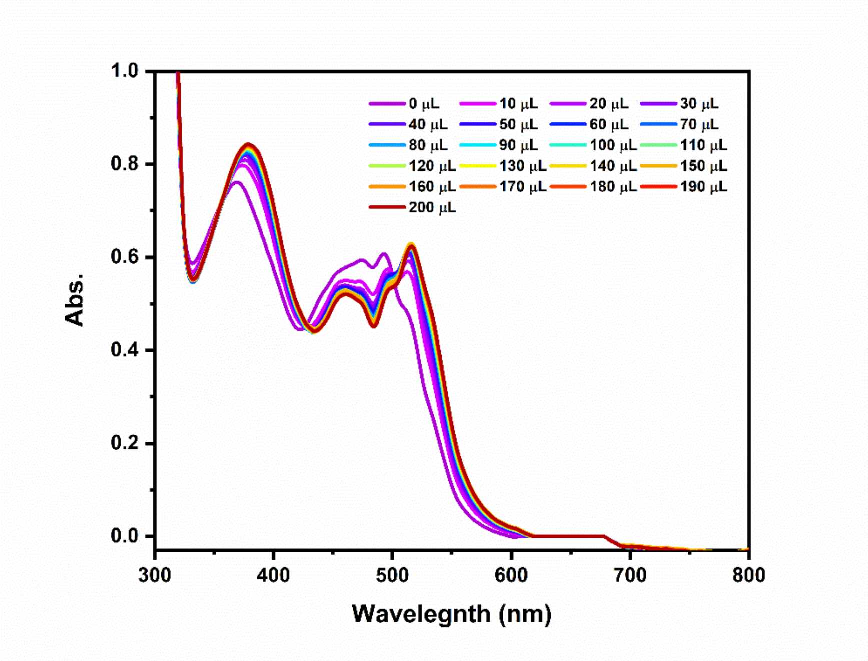

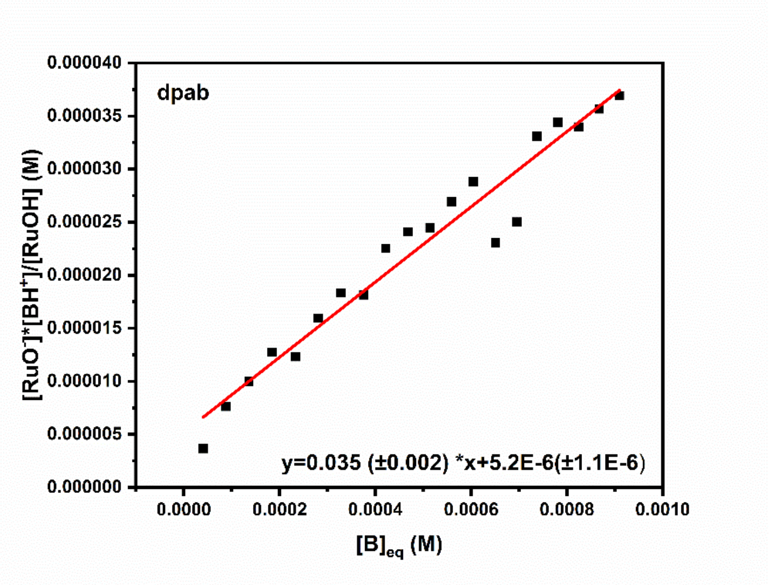

Figure S6. Steady-state UV-Vis titration with 2-aminobenzimidazole (top) and linear fit of concentration data derived from absorbance changes to determine the pK_a of $[Ru(dpab)_2(4,4'\text{-}dhbpy)]^{2+}$ (bottom).

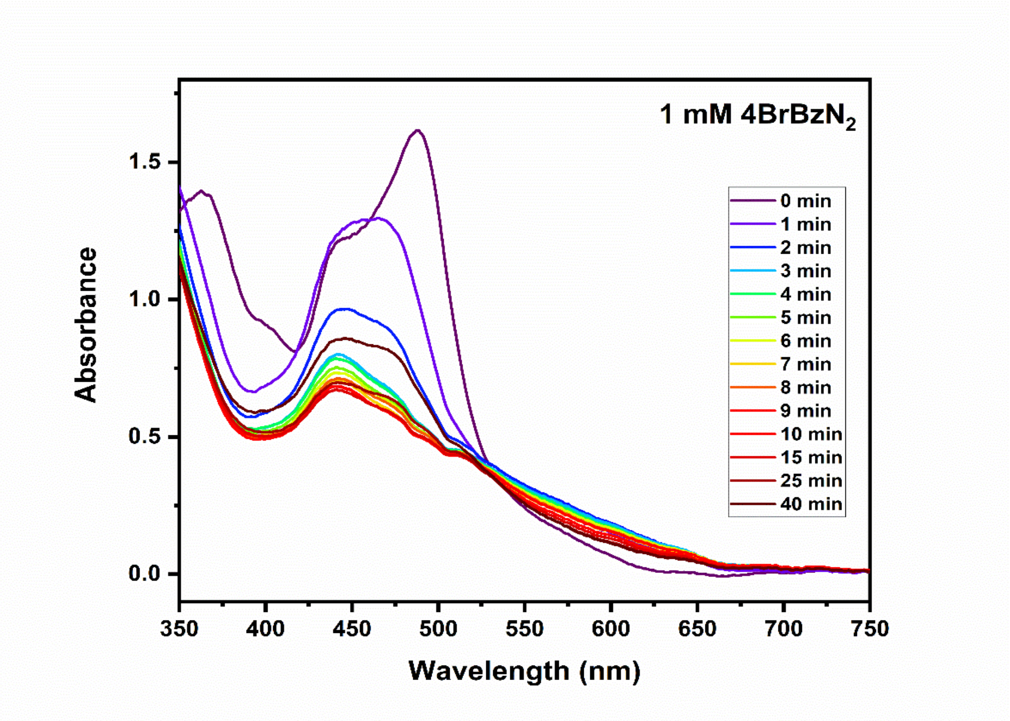

Figure S7. Absorption spectra of [(dpab)$_2$Ru(4,4'-dhbpy)]$^{2+}$ in CH_3CN in the presence of 1 mM 4-bromo-phenyldiazonium (BrBzN$_2$) (top) and 1 mM BrBzN$_2$ and 1 mM pyridine (bottom).

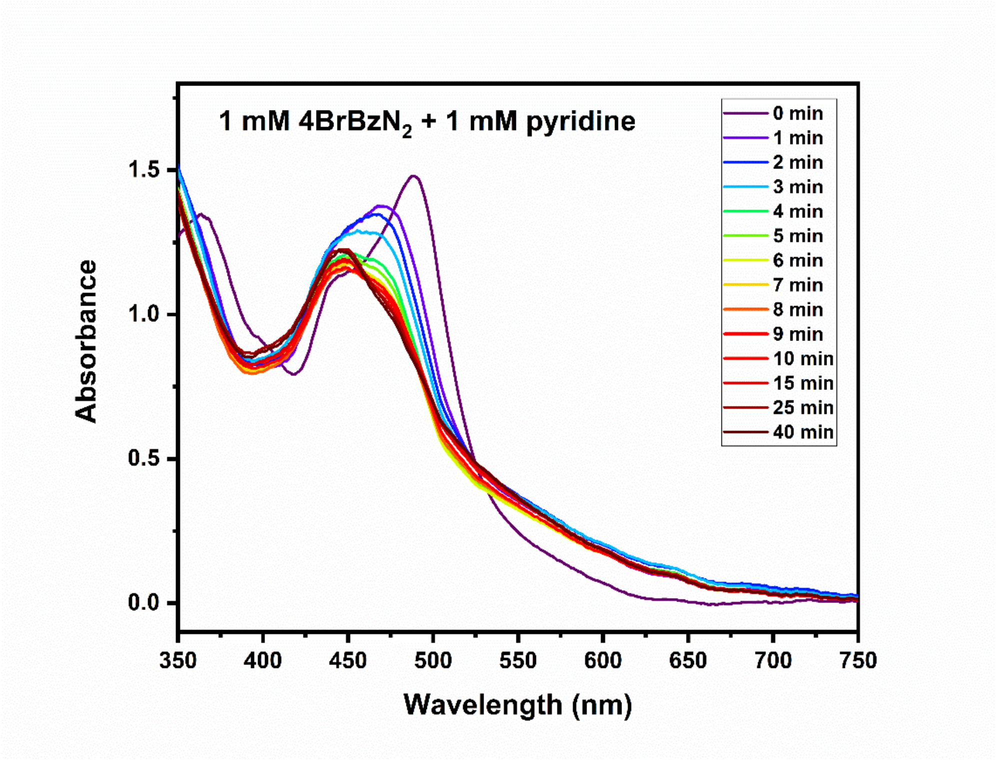

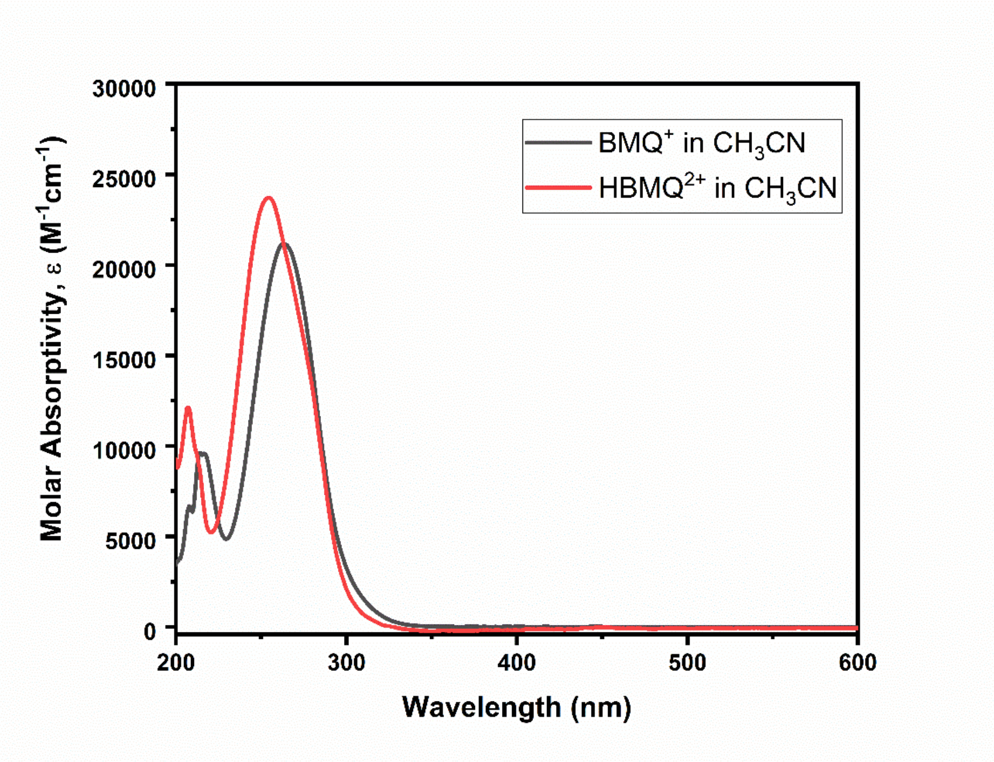

Figure S8. Absorption of BMQ^+ and $HBMQ^{2+}$ in CH_3CN

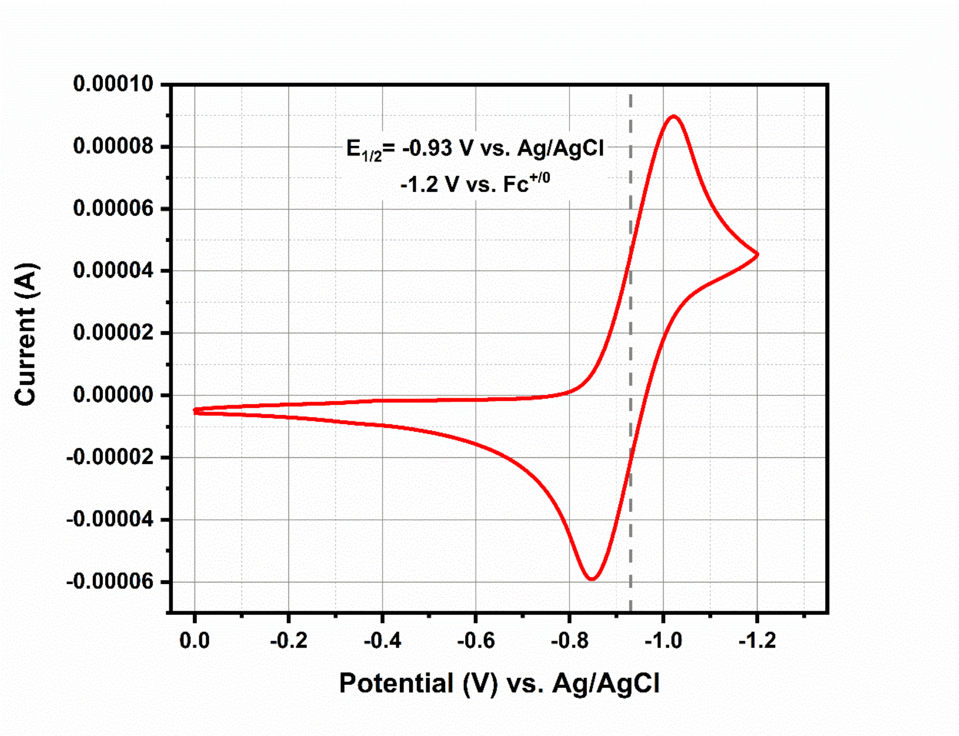

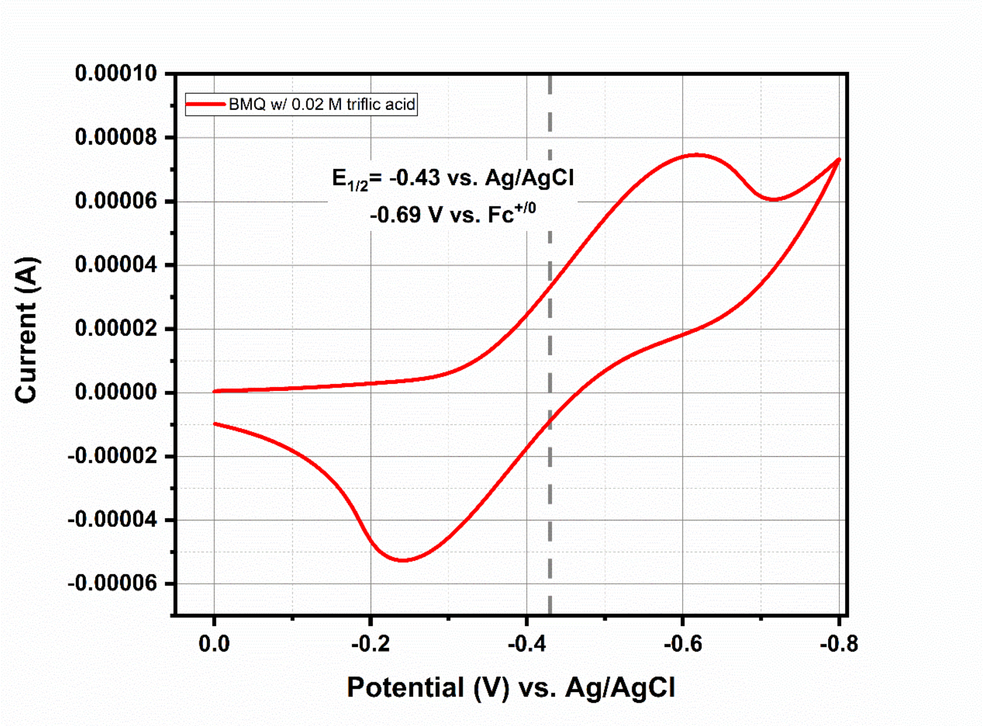

Figure S9. CV of BMQ^+ in CH_3CN and CV of BMQ^+ + 0.02 M triflic acid in CH_3CN

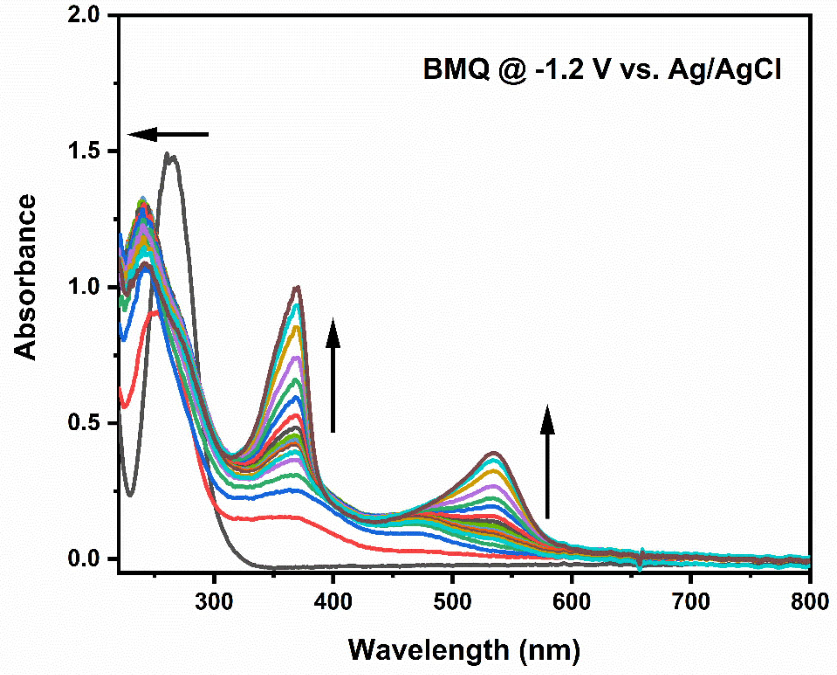

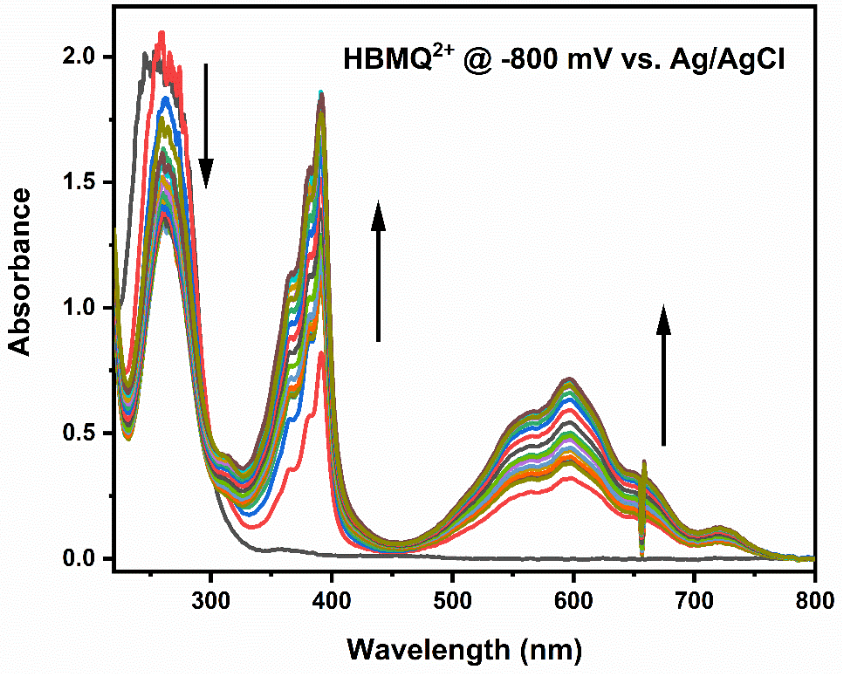

Figure S10. UV-vis spectra of $BMQ^{+}$and $HBMQ^{2+}$ upon one electron reduction in CH_3CN

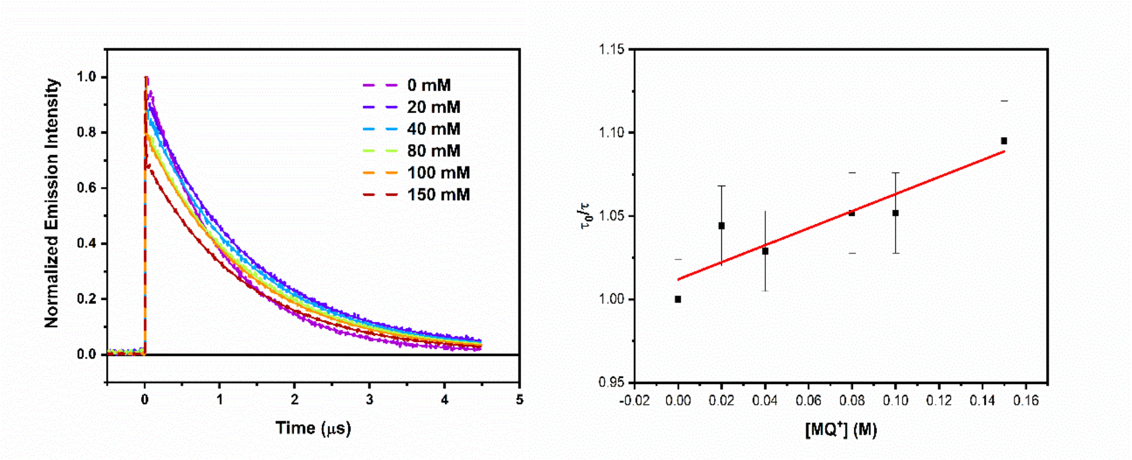

Figure S11. Luminescence lifetime quenching of $[Ru(dpab)_2(bpy)]^{2+}$ by MQ^+ (left) and the resulting Stern-Volmer plot (right)

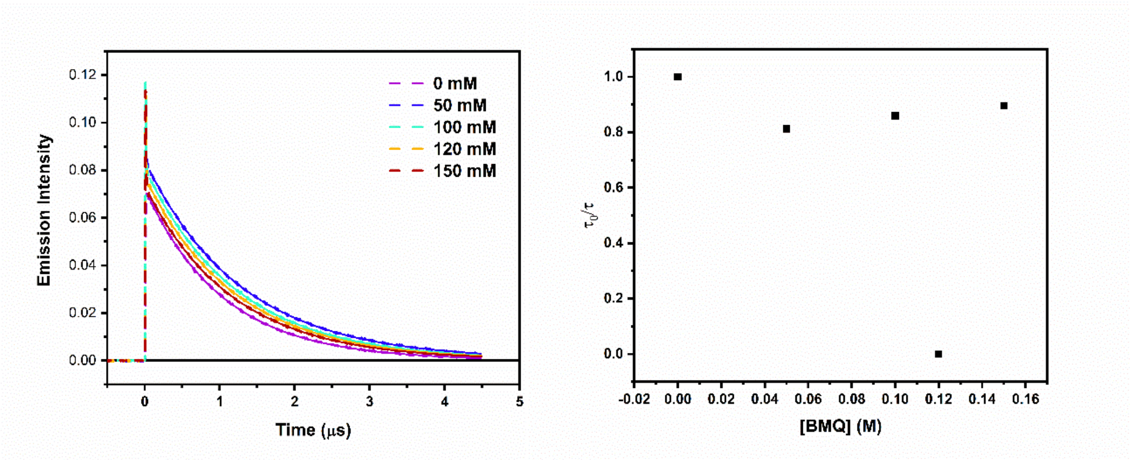

Figure S12. Luminescence lifetime quenching of $[Ru(dpab)_2(bpy)]^{2+}$ by BMQ^+ (left) and the resulting Stern-Volmer plot (right)

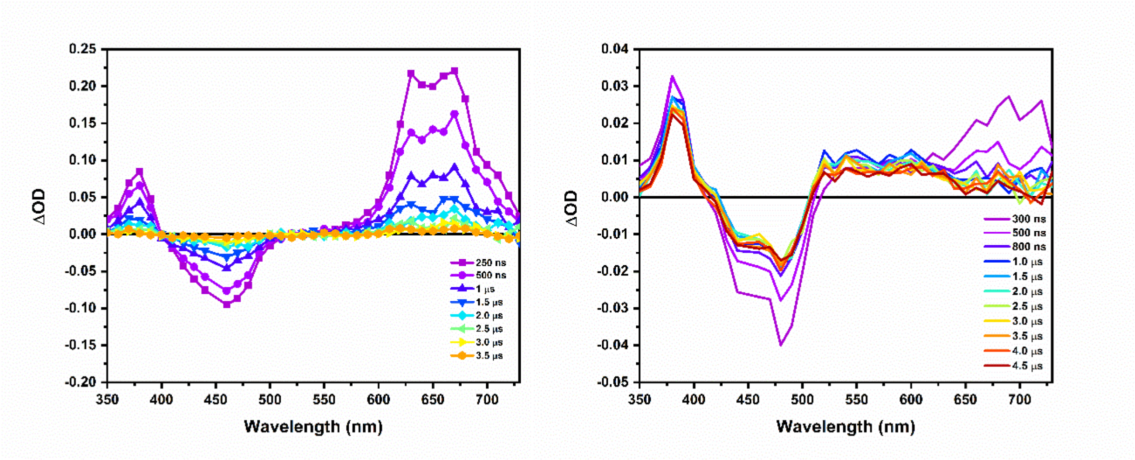

Figure S13. nsTA spectra of $[Ru(dpab)_2(4,4'\text{-dhbpy})]^{2+}$in the absence of MQ^+ (left) and in the presence of 140 mM MQ^+ (right).

NOTE: The excited state absorption between 600 and 700 nm is actually the observed signal when the recorded ΔOD is "corrected" by compensating for the excited state emission observed at any given wavelength. Unfortunately, the method used overcompensated for the emission that is unavoidably observed along with the transient absorption. The emission "maximum" and relative intensity are a function of a variety of experimental factors including the PMT voltage required to obtain reasonable absorption signal at a particular wavelength (related to the intensity of the pulsed white light source).

Table S1. Calculated Gibbs free energy for ground state reactions given in kcal/mol

	**MQ^+**			**BMQ^+**		
	**ΔG_{ET}**	**ΔG_{PT}**	**ΔG_{PCET}**	**ΔG_{ET}**	**ΔG_{PT}**	**ΔG_{PCET}**
$[Ru(bpy)_2(4,4'\text{-dhbpy})]^{2+}$	52.6	10.0	35.0	50.8	11.7	33.3
$[Ru(dpab)_2(4,4'\text{-dhbpy})]^{2+}$	54.5	7.4	43.4	52.6	9	41.7
$[Ru(bpy)_2(4,4'\text{-}(OMe)_2bpy)]^{2+}$	53.1			51.2		
$[Ru(dpab)_2(bpy)]^{2+}$	56.6			54.7		

4,4'-$(OMe)_2$bpy = 4,4'- dimethoxy-2,2'-bipyridine.

PT Reaction of Photoexcited [(dpab)$_2$Ru(4,4'-dhbpy)]$^{2+}$ and 3-acetylpyridine.*

The possibility of PT* was investigated for the reaction between [(dpab)$_2$Ru(4,4'-dhbpy)]$^{2+}$ and 3-acetylpyridine. 3-acetylpyridine, with a pK_a of 10.7 in acetonitrile, was chosen as a model base for MQ^+, being slightly more basic than MQ^+ (pK_a of 10.3).[16] Although the free energy for the proton exchange reaction (+2.3 kcal/mol) is similar to what is expected of MQ^+, electrostatic factors must also be considered. Because MQ^+ is a cation, there is a thermodynamic penalty for bringing together the like-charged species, however, in considering the overall proton transfer reaction, the separation of species after reaction is also important. Taking both formation of the encounter complex and separation of the proton transfer products into consideration, the net change in charge is zero, and therefore its contribution to the free energy for proton transfer is likely to be negligible. This is not the case for the model PT study with 3-acetylpyridine. As such, the free energy for excited-state proton transfer must include the calculation of the energy to bring together the dicationic Ru complex and the uncharged 3-acetylpyridine, and for the separation of the two monocationic species. An electrostatic work term can be included in the calculation of the free energy for proton transfer much like in photoinduced electron transfer reactions. **Equation 2-4** shows the formula used to calculate the free energy for excited-state proton transfer to 3-acetylpyridine.[22]

$$\Delta G_{PT} = -2.303RTln(\Delta pK_a) + N_A(W_P - W_R) \qquad \textbf{Equation 2}$$

$$W_P = \frac{z(A^-)z(BH^+)e^2}{4\pi\varepsilon_0\varepsilon_R a} \qquad \textbf{Equation 3}$$

$$W_R = \frac{z(AH)z(B)e^2}{4\pi\varepsilon_0\varepsilon_R a} \qquad \textbf{Equation 4}$$

In the expressions above, W_P and W_R are the work terms for product separation and formation of the encounter complex respectively, where z is the charge of the species, e is the elementary charge, ε_0 is the permittivity of vacuum (8.854 x 10^{-12} $C^2J^{-1}m^{-1}$), ε_r is the relative medium static permittivity (dielectric constant), and a is the distant between the two species). In the reaction between [(dpab)$_2$Ru(4,4'-dhbpy)]$^{2+}$ and 3-acetylpyridine, W_R is zero, and so only the term for product separation needs to be included. The distance between the metal complex and 3-acetylpyridine was calculated from the collision radii for the two species using the molecular mechanics optimized structures. Taking this into account, the calculated work term is -0.82 kcal/mol, making the free energy for proton transfer +1.5 kcal/mol.

The reaction of [(dpab)$_2$Ru(4,4'-dhbpy)]$^{2+}$ and 3-acetylpyridine was explored with transient absorption spectroscopy. 3-acetlypyridine and its conjugate acid do not absorb in the visible region of the spectrum, so the PT products are expected to match the simulated spectrum shown in figure 5. Stern-Volmer quenching analysis showed that PT* is slow for this system (k_q = 1.3 x 10^7 $M^{-1}s^{-1}$) , so concentrations of 3-acetylpyridine as high as 500 mM were used in TA analysis.. The transient absorption spectrum shown in Figure 7 shows the prominent absorption features that allow PT* products

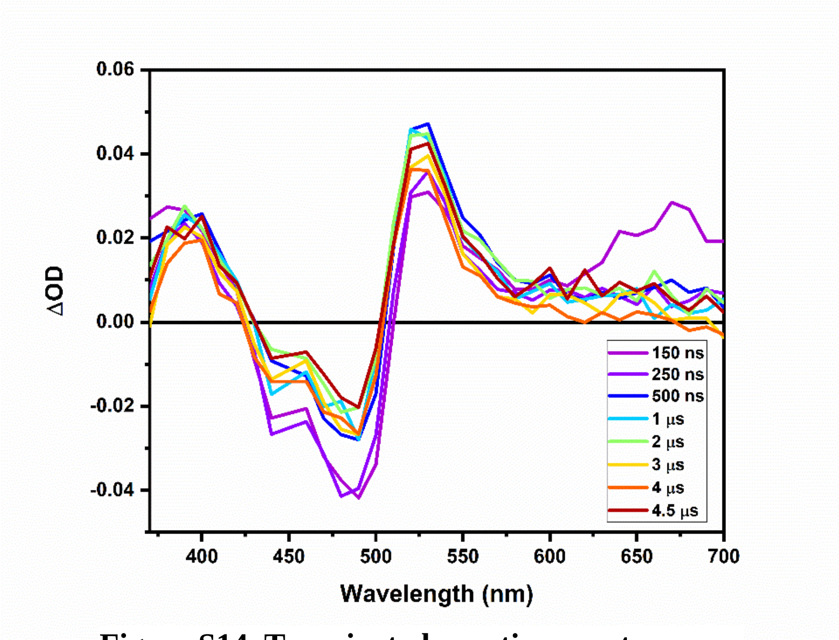

Figure S14. Transient absorption spectrum following 450 nm excitation of $[(dpab)_2Ru(4,4'\text{-}dhbpy)]^{2+}$ in the presence of 500 mM 3-acetylpyridine.

to be distinguished from ET* and PCET* products. The absorbance at around 530 nm is a distinguishing feature for the proton transfer products, along with the lower molar absorptivity absorption at 400 nm.

The proton transfer to 3-acetylpyridine is slightly less endergonic than that to MQ^+. Thus, PT* could be observed between $[(dpab)_2Ru(4,4'\text{-}dhbpy)]^{2+}$ and MQ^+ and it would be revealed by the TA spectrum.

Equations Used for Determination of Free Energies for PT*, ET* and PCET*

A. PT*: For calculating the free energy for proton transfer, the calculation is made by taking the difference in p*K*a value between the chromophore and the base, this gives the equilibrium constant for the proton transfer reaction, which can then be used to calculate the Gibbs free energy for proton transfer. This can be done for the ground state, excited state, and the one-electron oxidized state of the chromophore. For the proton transfer reaction, the work term for bringing the two charged species will be zero. Equations 5-7 show the calculation of the free energy for proton transfer including the reactant and product work terms. In these equations, *R* is the ideal gas constant, *T* is the temperature, Δp*K*a is the difference in p*K*a between the chromophore in either the ground state, excited state or one electron oxidized forms, and the quencher. N_A is Avogadro's number, W_P is the work terms for product separation, W_R is the work term for bringing together the reactants, ε_0 is the permittivity of vacuum constant, *a* is the distance between the two species, *z* is the charge on each compound, and *e* is the elementary charge.

$$\Delta G_{PT} = -2.303RTln(\Delta pK_a) + N_A(W_P - W_R) \qquad \textbf{(5)}$$

$$W_P = \frac{z(A-)z(BH+)e^2}{4\pi\ \varepsilon_0\ \varepsilon_R\ a} \qquad \textbf{(6)}$$

$$W_R = \frac{\mathrm{z(AH)z(B)}e^2}{4\pi\ \varepsilon_0\ \varepsilon_R\ a} \qquad \textbf{(7)}$$

Because the reactants will go from a 2+/1+ combination to products with a 1+/2+ charge combination for the pure proton transfer in either the ground or excited state, the work term will end up being zero. For proton transfer following electron transfer, the work term cannot be omitted, as W_P will be nonzero, while W_r will be zero, assuming the separation distance remains constant. The same equations for work can be applied to the electron transfer reaction and proton coupled electron transfer reactions, as will be discussed further below.

B. ET*: The free energy for the electron transfer can likewise be calculated using the redox potentials for the complexes and the quenchers. ΔG_{ET} can be calculated for the ground state and excited state electron transfer reactions using equation 8. This again includes the work term for bringing the charged species together along with the work required for charge separation. The pair will go from 2+/1+ for formation of the precursor complex to 3+/0 following electron transfer. The distance between the complex and the quencher has been estimated using a value of 6 Å for the ruthenium complex based on $[Ru(bpy)_3]^{2+}$ and the collision radius for the molecular mechanics optimized structure of the quenchers. From gas phase molecular mechanics calculations, the collision radii of MQ^+ and BMQ^+ were found to be 3.5 Å and 4.5 Å, respectively. In equation 8, *F* is the Faraday constant, *n* is the number of electrons being transferred, and $E^0(Ru^{III/II})$ and $E^0(MQ^{+/0})$ are the reduction potentials for the ruthenium(III) complex and bipyridinium quencher.

$$\Delta G_{ET} = -nF(E^0(Ru^{III/II}) - E^0(MQ^{+/0})) + N_A(W_P - W_R) \qquad \textbf{(8)}$$

For calculation of the free energy for excited state electron transfer, the oxidation potential of the excited state of the ruthenium complex ($E^0(Ru^{III/II})$- E_{em}) is substituted in for $E^0(Ru^{III/II})$).

C. PCET*: For calculation of the free energy for proton-coupled electron transfer, two methods can be used, and should yield the same result within a margin of error attributable to the error associated with individual experimental values. The first method involves taking the sum of the free energy for PT*, followed by ET* from the deprotonated complex to the protonated quencher. The second

method involves the sum of free energies for ET* and ground state proton transfer from the Ru^{III} to the reduced quencher. The reason for this is that excited state electron transfer deactivates the excited state, such that any proton transfer subsequently should occur from the ground state potential surface. The more reliable estimates come from the first approach, since determination of the pKa values for the Ru^{III} hydroxybipyridine complex and the reduced MQ^{+} (MQ^{0}) have large margins of error.
